# Corresponding morphological and molecular indicators of crude oil toxicity to the developing hearts of mahi mahi

**DOI:** 10.1038/srep17326

**Published:** 2015-12-10

**Authors:** Richard C. Edmunds, J. A. Gill, David H. Baldwin, Tiffany L. Linbo, Barbara L. French, Tanya L. Brown, Andrew J. Esbaugh, Edward M. Mager, John Stieglitz, Ron Hoenig, Daniel Benetti, Martin Grosell, Nathaniel L. Scholz, John P. Incardona

**Affiliations:** 1 National Research Council Associate Program, under contract to Northwest Fisheries Science Center, National Marine Fisheries Service, NOAA, 2725 Montlake Blvd. E., Seattle, WA 98112 USA; 2 Frank Orth and Associates, under contract to Northwest Fisheries Science Center, National Marine Fisheries Service, NOAA, 2725 Montlake Blvd. E., Seattle, WA 98112 USA; 3 Environmental and Fisheries Science Division, Northwest Fisheries Science Center, National Marine Fisheries Service, NOAA, 2725 Montlake Blvd. E., Seattle, WA 98112 USA; 4 Department of Marine Science, University of Texas, Marine Science Institute, 750 Channel View Dr., Port Aransas, TX 78373 USA; 5 Department of Marine Biology and Ecology, University of Miami, Rosenstiel School of Marine and Atmospheric Science, 4600 Rickenbacker Cswy., Miami, FL 33149 USA

## Abstract

Crude oils from distinct geological sources worldwide are toxic to developing fish hearts. When oil spills occur in fish spawning habitats, natural resource injury assessments often rely on conventional morphometric analyses of heart form and function. The extent to which visible indicators correspond to molecular markers for cardiovascular stress is unknown for pelagic predators from the Gulf of Mexico. Here we exposed mahi (*Coryphaena hippurus*) embryos to field-collected crude oil samples from the 2010 Deepwater Horizon disaster. We compared visible heart defects (edema, abnormal looping, reduced contractility) to changes in expression of cardiac-specific genes that are diagnostic of heart failure in humans or associated with loss-of-function zebrafish cardiac mutants. Mahi exposed to crude oil during embryogenesis displayed typical symptoms of cardiogenic syndrome as larvae. Contractility, looping, and circulatory defects were evident, but larval mahi did not exhibit downstream craniofacial and body axis abnormalities. A gradation of oil exposures yielded concentration-responsive changes in morphometric and molecular responses, with relative sensitivity being influenced by age. Our findings suggest that 1) morphometric analyses of cardiac function are more sensitive to proximal effects of crude oil-derived chemicals on the developing heart, and 2) molecular indicators reveal a longer-term adverse shift in cardiogenesis trajectory.

It is well established that crude oil exposures cause a consistent syndrome of cardiac-related developmental defects in fish. This cardiotoxicity is attributable to the soluble fraction of polycyclic aromatic hydrocarbons (PAHs), particularly those containing three rings such as the phenanthrenes, flourenes, and dibenzothiophenes^1^,^2^,^3^,^4^. These PAHs disrupt the normal function and morphogenesis of the heart^3^,^4^ via the blockade of potassium and calcium conductances that underpin normal excitation-contraction (E-C) coupling in individual heart muscle cells (cardiomyocytes^5^). Canonical crude oil cardiotoxicity is characterized by abnormalities in heart muscle contractility, heart rhythm, and circulation. Relatively severe circulatory defects often lead to heart failure, as evident from fluid accumulation (edema) in the pericardial space. Pericardial edema was demonstrated in pink salmon and Pacific herring exposed to crude oil from the 1989 Exxon Valdez oil spill^6^,^7^, and has since been documented in many freshwater and marine fish species exposed to crude oils from distinct geological sources worldwide^8^,^9^,^10^,^11^,^12^. Edematous embryos and larvae commonly have concurrent craniofacial (eye and jaw) defects and body axis deformities that are a downstream consequence of improper embryonic cardiogenesis^4^.

The crude oil cardiotoxicity syndrome in fish is traditionally assessed using conventional light and digital video microscopy. These methods have proven robust in terms of quantifying both functional and morphological impacts on the developing heart (e.g.,^8^,^9^,^13^). However, digital imaging is relatively labor intensive and therefore a challenge for large replicate samples from rapidly developing warm water species. Moreover, live imaging is often not an option for destructively sampled embryos and larvae from oiled habitats (e.g., from shipboard plankton tows). Lastly, fish that survive embryonic crude oil exposures, and appear outwardly normal as juveniles, nevertheless suffer delayed mortality^14^,^15^ or reduced swimming performance^16^. Delayed mortality has recently been attributed to form following function during heart morphogenesis, wherein transient and sublethal impairments in heart muscle contractility produce lasting negative outcomes for heart shape and cardiac performance^17^,^18^. This raises the possibility of subtle but important forms of injury in fish that do not develop visually overt pericardial edema as embryos and larvae in response to crude oil-derived PAHs.

Biomarkers such as cytochrome P450 1A (CYP1A) have provided evidence of crude oil exposure in fish for decades, in both laboratory and field studies, at both molecular (e.g., *cyp1a* gene expression) and biochemical (e.g., 7-ethoxyresorufin-O-deethylase, or EROD enzymatic activity) levels^19^,^20^. The CYP1A pathway provides metabolic protection against toxic chemicals that are ligands for the aryl hydrocarbon receptor (AhR), and the AhR-mediated up-regulation of *cyp1a* is a very sensitive indicator of exposure to certain PAHs. However, corresponding molecular biomarkers that are phenotypically anchored to cardiac injury have yet to be identified for fish early life stages. In theory, such indicators might reveal toxicity in oil-exposed fish with ostensibly normal heart shapes and rhythms. They could also improve high-throughput screening and refine *in situ* health assessments for wild fish species that spawn in oiled habitats.

The identification and validation of new markers for cardiac dysregulation during early heart development poses some important challenges. First, depending on the species, cardiogenesis may progress rapidly, on a timescale of hours to a few days. During this window (e.g., 12–120 hpf for zebrafish) the normal expression patterns of cardiac-related genes is highly dynamic (reviewed by^21^). This creates problems of shifting baselines and low signal-to-noise ratios. For example, whereas a PAH exposure indicator such as *cyp1a* may be stably expressed at relatively low levels and then robustly upregulated in response to AhR activation (e.g.,^22^), the expression of genes responsive to cardiovascular stress may be altered against a more nuanced backdrop of up- or down-regulation as part of normal heart development (reviewed by^21^). Second, candidate molecular markers may be proximally or peripherally related to the primary effects of PAHs on heart muscle cells (i.e., pharmacological blockade of E-C coupling). This might yield molecular indicators that are very sensitive to relatively non-specific aspects of the injury phenotype, such as inflammation or edema.

In the present study we compare conventional and molecular aspects of the crude oil cardiotoxicity syndrome in pelagic mahi mahi (*Coryphaena hippurus*). Mahi mahi are a recreationally and commercially important species in the northern Gulf of Mexico, and are thus included in the ongoing natural resource damage assessment (NRDA) in the aftermath of the 2010 Deepwater Horizon (DWH) oil spill, also known as Mississippi Canyon 252 (MC252). They spawn in the open ocean^23^, producing fertilized embryos that develop rapidly in upper surface waters. The 2010 spawning season overlapped in space and time with surface oil from the Deepwater Horizon spill^24^. Captive mahi mahi broodstock can be environmentally conditioned to spawn volitionally at land-based facilities^16^, making it possible to characterize the developmental toxicity of field-collected crude oil samples. This approach was recently used with several MC252 crude oil samples to compare thresholds for larval lethality in mahi mahi to those for earlier appearing cardiotoxic effects manifested as edema and reduced atrial contractility^25^. Here, we document the effects MC252 crude oil exposure on expression of candidate genes with known associations to contractility defects and other contributors to heart failure in vertebrates, notably humans. We also provide more complete functional and morphometric analyses of heart development to refine the phenotypic context for anchoring changes in cardiac gene expression. We then compared the relative sensitivities of these candidate molecular injury markers to the visible signs of crude oil cardiotoxicity.

## Results

### WAF preparations allowed exposure across a range of PAH concentrations and mixture composition

**Figure 1:**
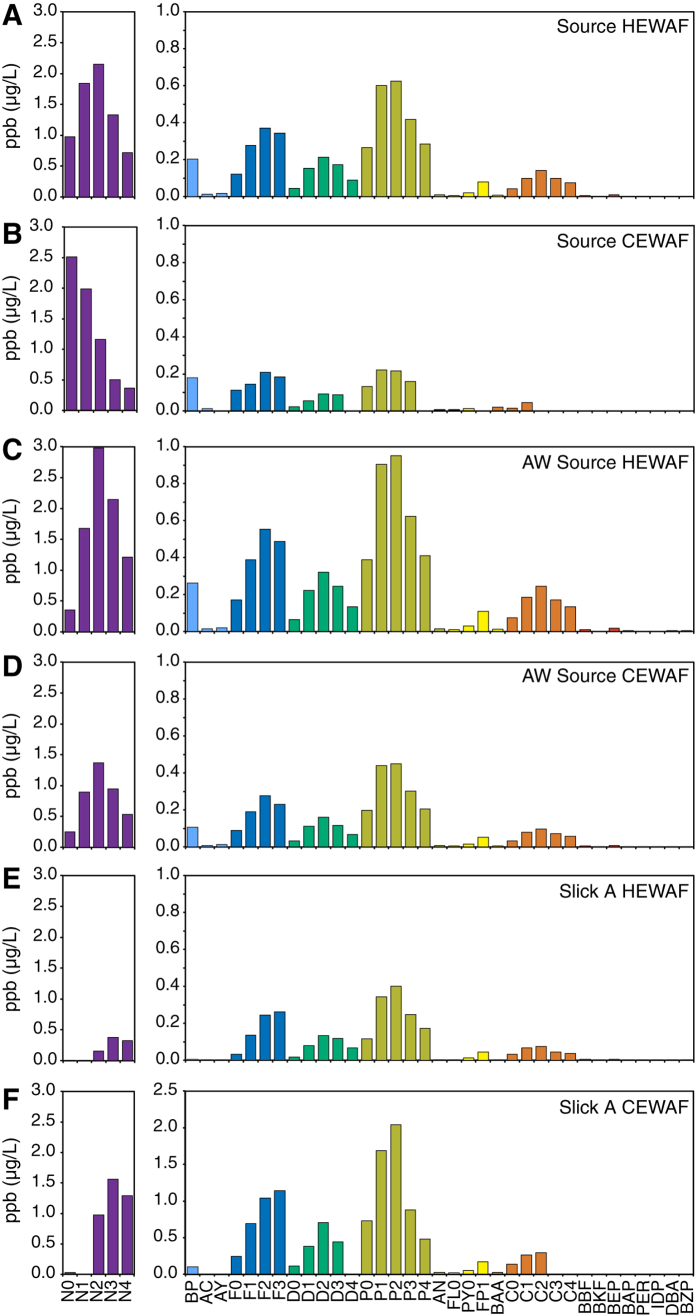
PAH concentrations in HEWAF and CEWAF preparations at the onset of mahi mahi embryo exposures. Values represent single measurements from the highest exposure levels for (**A**) Source HEWAF, (**B**) Source CEWAF, (**C**) AW-Source HEWAF, (**D**) AW-Source CEWAF, (**E**) Slick A HEWAF, and (**F**) Slick A CEWAF. N, naphthalenes; BP, biphenhyl; AC, acenaphthene; AY, acenaphthylene; F, fluorene; D, dibenzothiophene; P, phenanthrene; AN, anthracene; FL, fluoranthene; PY, pyrene; FP, fluoranthenes/pyrenes; BAA, benz[a]anthracene; C, chrysene; BBF, benzo[b]fluoranthene; BKF, benzo[j]fluoranthene/benzo[k]fluoranthene; BEP, benzo[e]pyrene; BAP, benzo[a]pyrene; PER, perylene; IDP, indeno[1,2,3-cd]pyrene, DBA, dibenz[a,h]anthracene/dibenz[a,c]anthracene; BZP, benzo[*ghi*]perylene. Parent compound is indicated by a 0 (e.g., N0), while numbers of additional carbons (e.g. methyl groups) for alkylated homologs are indicated as N1, N2, etc.

Total PAHs (particulate plus dissolved) were measured for high energy water accommodated fraction (HEWAF) and chemically enhanced water accommodated fraction (CEWAF) dilutions used in the exposure assays with each MC252 oil sample (Source, Artificially Weathered Source (AW-Source), Slick A; Fig. 1, Table 1). We report the sum of 50 PAHs (∑PAH50) routinely measured for the DWH NRDA as well as the sum of 40 PAHs (ΣPAH40) and the sum of the tricyclic PAH fraction (ΣTAH; fluorenes, dibenzothiophenes, and phenanthrenes) for comparison with previous studies (Table 1). The ∑PAH40 and ∑TAH ranged from 0.4 to 20.3 μg/L and 0.1 to 6.8 μg/L, respectively. Based on modeling from separate experiments, the dissolved fraction of PAHs was estimated for each assay (Table 1 and^25^). Generally, the less viscous Source and AW-Source samples were more readily dispersed either mechanically (HEWAF) or chemically with the dispersant Corexit 9500 (CEWAF), yielding exposures over a broader range of PAH concentrations (Table 1 and^25^). The compositions of the PAH mixtures in WAFs were consistent with previous studies^9^ and the compositions of the parent oil samples, with higher and lower concentrations of naphthalenes in Source oil WAFs compared to the more highly weathered Slick A, respectively (Fig. 1). These PAH concentrations and patterns were comparable to those measured in water samples collected during the active spill phase in the upper pelagic zone of a 96,000 km^2^ area centered around the damaged wellhead^9^.

### Edema accumulation was the primary morphological response of mahi mahi embryos exposed to MC252 crude oil

**Figure 2:**
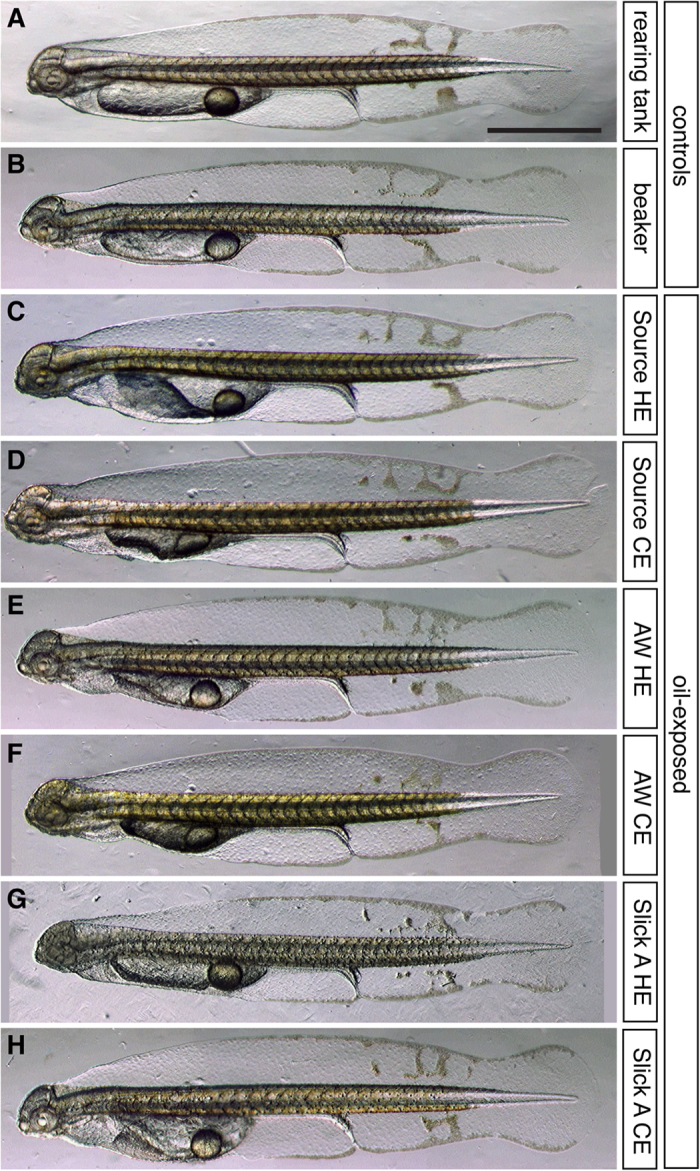
Gross morphology of hatching stage mahi mahi larvae exposed to MC252 HEWAF and CEWAF oil during embryonic development. Embryos were exposed from shortly after fertilization to at least ~12 h after hatching (i.e., 48–60 hpf). (**A,B**) Unexposed controls incubated in clean water in a hatchery rearing tank (A) or 1-L beaker (**B**). (**C–H**) Representative larvae from the highest exposure levels. (**C,D**) Source HEWAF (0.25% WAF, 20.3 μg/L ΣPAH) and CEWAF exposed (1.4% WAF, 8.5 μg/L ΣPAH). (**E,F**) AW-Source HEWAF (0.17% WAF, 14.6 μg/L ΣPAH) and CEWAF exposed (0.25% WAF, 10.5 μg/L ΣPAH). (**G,H**) Slick A HEWAF (0.15% WAF, 5.1 μg/L ΣPAH) and CEWAF exposed (9.13% WAF, 16.4 μg/L ΣPAH). *HE*, HEWAF; *CE*, CEWAF; scale bars, 1 mm.

Exposure of mahi mahi embryos to each MC252 oil sample and WAF type resulted in a primarily cardiotoxic phenotype (Fig. 2), aspects of which are also reported elsewhere^25^. Grossly, hatching stage larvae showed no morphological defects (see below) other than accumulation of edema involving the pericardial area and yolk sac. The occurrence of edema ranged from 29% to 90% at the most concentrated WAF dilutions tested, and while generally correlating with measured PAH concentrations (Supplemental Materials: Figure S1A), concentration-response curves for predicted ∑TAH concentrations (Supplemental Materials: Figure S1C) had greater overlap than those for either measured ∑PAH or predicted dissolved ∑PAH (Supplemental Materials: Figure S1B). During this early window of larval development, the still-forming head continues to elongate and rotate upward, while the yolk moves more posteriorly. The heart occupies a position between the head and the anterior of the yolk mass, resulting in a roughly triangular pericardial space surrounding the heart. In unexposed mahi mahi larvae, a very thin space typically extends posteriorly toward the endogenous, lipid-containing oil globule that is normally present in most pelagic fish eggs. This space is filled with red blood cells returning to the heart and will eventually become the cardinal vein (Supplemental Materials: Figure S2A). As fluid accumulates in oil-exposed fish and they become edematous, the pericardial space enlarges and forces the yolk mass posteriorly, thereby expanding the region of the developing cardinal vein (Supplemental Materials: Figure S2A’).

### Morphometric analyses of functional and morphological cardiotoxicity endpoints

**Figure 3:**
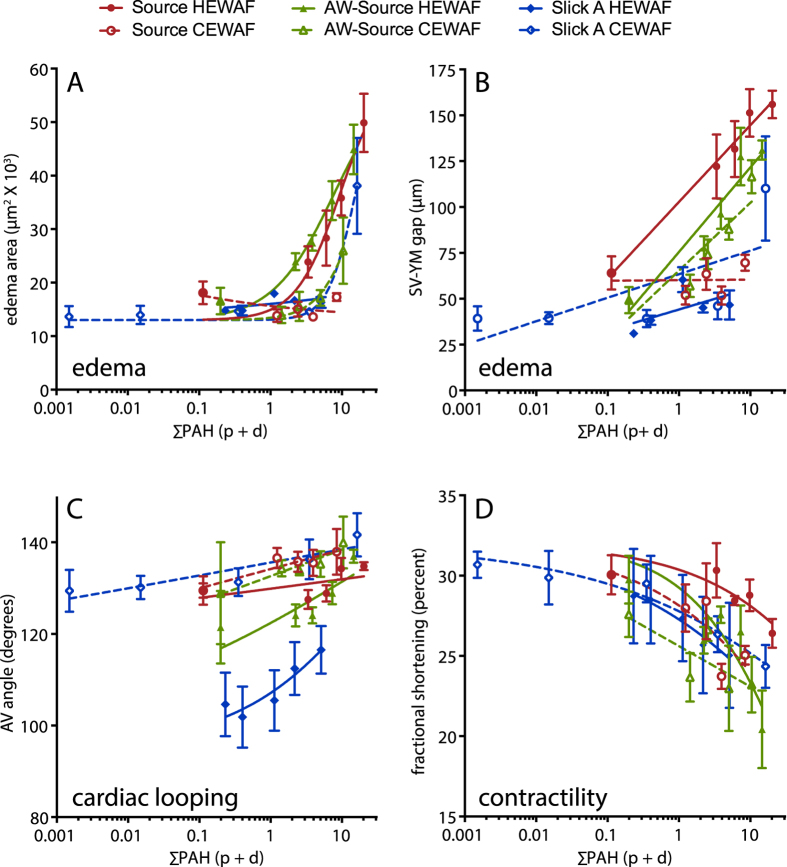
Morphometric indicators were generally dependent on PAH concentrations. Edema area (**A**) and sinus venosus–yolk mass (SV–YM) gap (**B**) were quantitative measures related to the severity of edema, atrioventricular (AV) angle (**C**) was a measure of heart morphology (chamber looping), and fractional shortening (**D**) was a functional measure of contractility based on chamber diameters. All measures were made in digital video frames (see Methods) and plotted with lines representing non-linear (**A,D**) or linear regression (**B,C**) models. As described in the text, these regressions were used for graphical representation only and not statistical analyses (e.g., Figs 4 and 5, Supplemental Materials: Table S1). ∑PAH40 concentrations represent actual measured values of HEWAF dilutions including both particulate whole oil and dissolved PAHs. Data are mean ± SEM.

In addition to a binary score of presence/absence of pericardial or yolk sac edema^25^, morphometric analyses focused on several aspects of cardiac development and function. These included quantification of the 2-dimensional area occupied by edema fluid (edema area; Supplemental Materials: Figure S2A, A’) and the linear distance between the posterior edge of the sinus venosus and the anterior edge of the yolk mass (SV-YM gap; Supplemental Materials: Figure S2B, B’), both of which provide a continuous measure related to the severity of edema. The angle between the atrial and ventricular chambers was used to assess looping of the cardiac chambers, a process that ultimately brings the atrium and ventricle into an adjacent arrangement (AV angle; Supplemental Materials: Figure S2C, C’). An increased AV angle is indicative of poor looping. Finally, atrial contractility was measured as fractional shortening based on the diameter of the atrium during the diastolic (relaxed) and systolic (contracted) phases (see Methods). In most tests there were concentration-dependent effects on each of these morphometric endpoints based on measured ∑PAH40 (Fig. 3; graphical representation only, statistical analysis provided below). Importantly, PAH concentration ranges spanned by each test were generally low (<20 μg/L) and endpoints were below a 50% effect level. There was no effect of oil exposure on heart rate (Supplemental Materials: Figure S3). Embryos exposed to the dispersant Corexit 9500 alone showed no differences from controls for any cardiotoxicity measure (Supplemental Materials: Figure S3).

### Candidate molecular indicators for cardiotoxicity

We used quantitative real-time PCR (qPCR) to assess the responses of ten genes involved in cardiac development and function to MC252 crude oil exposure (Table 2). Additional extracardiac genes served to assess other potential effects on development or as normalizing reference genes. While we based gene selection on known functions (primarily in zebrafish) or roles in human heart failure pathways, the final suite of genes was determined by the ability to reliably quantify their expression in mahi mahi. Candidate genes included *gata4*, *nkx2.5*, and *tbx5*, which are key transcription factors involved in cardiomyocyte determination and differentiation^21^; the atrial- and ventricle-specific structural myosin heavy chain subunits *amhc* and *vmhc*, respectively; the regulatory cardiac myosin light chain *cmlc2*^21^; atrial and B-type natriuretic peptides *nppa* and *nppb*, which are homeostatic regulators of contractility and are up-regulated in human heart failure^26^; and the four-and-a-half LIM domain family protein 2 (*fhl2*), which functions in regulation of cardiomyocyte elasticity^27^, and is associated with abnormal hypertrophy and heart failure when down-regulated^28^,^29^. Specificity of cardiac expression for the most robustly perturbed genes was verified by whole-mount *in situ* hybridization in larvae (Supplemental Materials: Figure S4A), while all candidate molecular indicators were tested for ventricular expression in adult heart by qPCR (Supplemental Materials: Figure S4B). To assess potential non-specific, general effects on development and other pathophysiological responses, we quantified the expression of extracardiac gene *ikaros*, which encodes a transcription factor required for embryonic and early larval lymphopoiesis^30^ and serves as a marker of inflammatory responses in fish^31^. Additionally, the mahi mahi ortholog of heat shock protein gene *hsp70* was assessed as a general stress response marker. Finally, orthologs of the PAH-inducible cytochrome P450 genes *cyp1a* and *cyp1b1* were measured as indicators of PAH exposure. Reference genes used for normalization included the cardiac-specific alpha actin 1b gene *actc1b*, and the broadly-distributed ribosomal protein subunit gene *rps25*.

In almost all cases, cardiotoxicity indicator genes were down-regulated in response to MC252 oil exposure. The genes that consistently had the largest responses were related to myofiber structure and contractility, including *amhc*, *cmlc2*, *fhl2, nppb*, and *vmhc*. In one test, Slick A CEWAF, *gata4* was modestly up-regulated, whereas it was modestly down-regulated in Source and AW-Source CEWAF assays or not affected (i.e., all three HEWAF assays).

### Correlation of concentration-responsiveness for cardiac morphometric and molecular indicators

**Figure 4:**
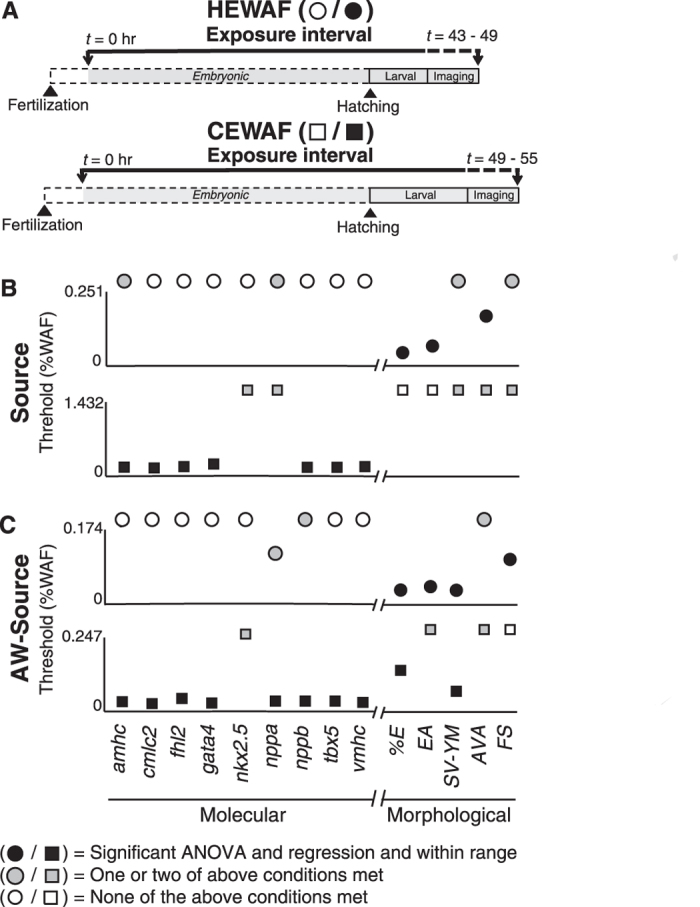
Comparison of threshold %WAF concentrations across molecular and morphometric cardiotoxicity indicators determined by linear regression for Source and AW-Source oils. (**A**) Schematic depicting experimental design showing length of exposure, time of hatching, and period of imaging/tissue collection for HEWAF and CEWAF experiments. (**B**) Threshold %WAF values following exposure to Source 948 HEWAF (0–0.251%; top) and CEWAF (0–1.432%; bottom) preparations. (**C**) Threshold %WAF values following exposure to AW-Source oil HEWAF (0–0.174%; top) and CEWAF (0–0.247%; bottom) preparations. Molecular (*amhc, cmlc2, fhl2, gata4, nkx2.5, nppa, nppb, tbx5, vmhc*) and morphological (%E, EA, SV-YM, AVA, FS; see Methods) indicators are presented on left and right of X-axis break, respectively. Circles, HEWAF; squares, CEWAF; filled black symbols met all three of the following conditions: 1) significant one-way ANOVA (*p* < 0.05), 2) significant log-linear regression (*p* < 0.05), and 3) threshold %WAF within exposure range empirically tested (e.g., Supplemental Materials: Figure S5A); filled gray symbols met one or two of the above conditions; open symbols met none of the above conditions. Indicators with symbol above Y-axis maximum denotes non-significant log-linear regression (*p* > 0.05) or threshold %WAF value outside range empirically tested.

**Figure 5:**
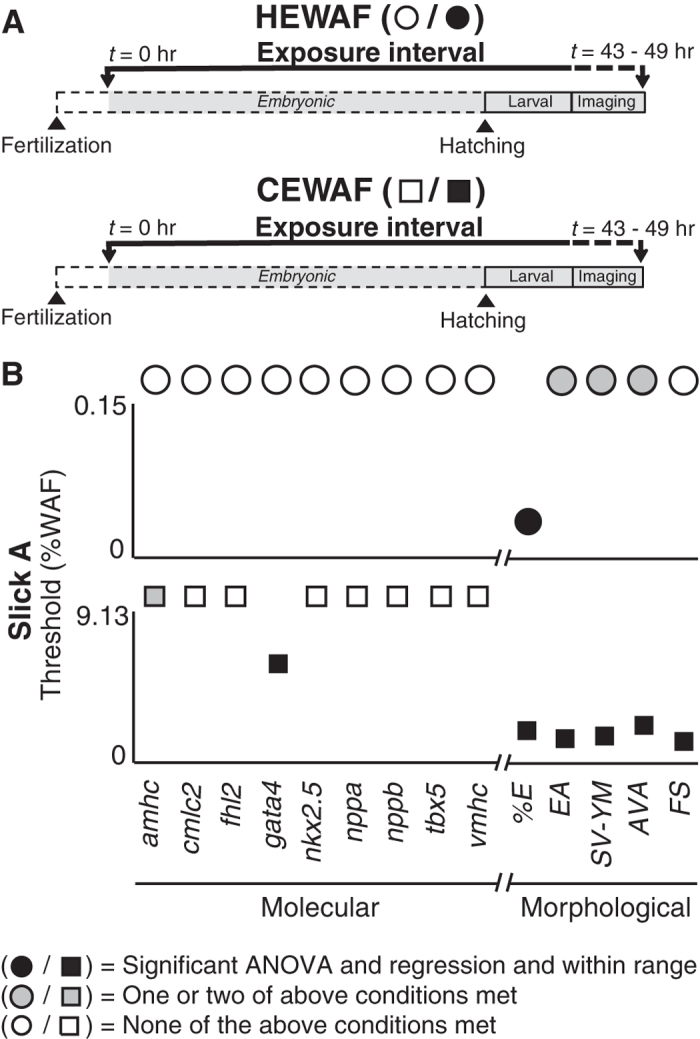
Comparison of threshold %WAF concentrations across molecular and morphometric cardiotoxicity indicators determined by linear regression for Slick A oil. (**A**) Schematic depicting experimental design showing length of exposure, time of hatching, and period of imaging/tissue collection for HEWAF and CEWAF experiments. (**B**) Threshold %WAF values following exposure to Slick A HEWAF (0–0.15%; top) and CEWAF (0–9.13%; bottom) preparations. Molecular (*amhc, cmlc2, fhl2, gata4, nkx2.5, nppa, nppb, tbx5, vmhc*) and morphological (%E, EA, SV-YM, AVA, FS; see Methods) indicators are presented on left and right of X-axis break, respectively. Circles, HEWAF; squares, CEWAF; filled black symbols met all three of the following conditions: 1) significant one-way ANOVA (*p* < 0.05), 2) significant log-linear regression (*p* < 0.05), and 3) threshold %WAF within exposure range empirically tested (e.g., Supplemental Materials: Figure S5A); filled gray symbols met one or two of the above conditions; open symbols met none of the above conditions. Indicators with symbol above Y-axis maximum denotes non-significant log-linear regression (*p* > 0.05) or threshold %WAF value outside range empirically tested.

Concentration-response data for both morphometric and candidate molecular indicators were analyzed by both one-way Analysis of Variance (ANOVA) and log-linear regression (Supplemental Materials: Table S1). Previously, ANOVA only was used to compare edema and atrial contractility to lethality data^25^. Linear regressions utilized WAF dilution factors (Supplemental Materials: Figure S6; see Methods) so that effect threshold values could be subsequently estimated for different PAH metrics (e.g. ∑PAH vs. sum of tricyclic families only). Some tests showed significant effects of oil exposure by ANOVA but not linear regression, and *vice versa*. However, the indicators considered the most robust (Fig. 4) were those that met the following three conditions: 1) a significant response by one-way ANOVA (*p* < 0.05), 2) a significant response by log-linear regression (i.e., slope ≠ 0, *p* < 0.05), and 3) a calculated response threshold that fell within the range of empirically tested PAH concentrations. For exposures with Source and AW-Source oil samples, HEWAF and CEWAF assays were paired using the same controls, with data collection and RNA sampling performed on the same day with HEWAF and CEWAF assay data collection/sampling occurring in the morning and afternoon, respectively, so that some samples were separated by almost 12 hours (Fig. 4A). Using the above conditions for endpoint robustness, concentration-dependency was observed for 3/5 morphometric indicators and no molecular indicators in the Source HEWAF exposure (Fig. 4B, top; dissolved ∑PAH40 0.1–15.1 μg/L), and no morphometric indicators but 7/9 molecular indicators in the Source CEWAF exposure (Fig. 4B, bottom; dissolved ∑PAH40 0.1–7.3 μg/L). For AW-Source oil, concentration-dependency was observed for 4/5 morphometric indicators and no molecular indicator in the HEWAF exposure (Fig. 4C, top; dissolved ∑PAH40 0.1–6.8 μg/L), and 2/5 morphometric indicators and 8/9 molecular indicators in the CEWAF exposure (Fig. 4C, bottom; dissolved ∑PAH40 0.1–5.0 μg/L). HEWAF and CEWAF assays with Slick A were carried out on separate days, and imaging/tissue collection for both tests occurred in the same time frame (43–49 h) as the Source and AW-Source HEWAF assays (Fig. 5A). For the Slick A HEWAF exposure (Fig. 5B) only a single morphometric indicator (edema binary score) was robustly concentration-dependent, consistent with this assay having the lowest exposure concentrations (dissolved ∑PAH40 0.2–2.6 μg/L). In contrast, the Slick A CEWAF exposure (Fig. 5B, bottom; dissolved ∑PAH40 0–5.3 μg/L) resulted in concentration-dependency for 5/5 morphometric indicators and a single molecular indicator (*gata4* up-regulation).

In all tests there were endpoints that did not meet all three conditions (see above), but were nevertheless significant by one-way ANOVA or log-linear regression. In Source and AW-Source HEWAF and CEWAF tests some endpoints were significant by both ANOVA and linear regression, but had calculated threshold values above the range of tested PAH concentrations (Supplemental Materials: Table S1). For Source HEWAF these endpoints included atrial contractility, while Source CEWAF included SV-YM gap (Fig. 4B). For AW-Source HEWAF these endpoints included AV angle and *nppb* levels, while AW-Source CEWAF included edema area and AV angle (Fig. 4C). Additionally, some endpoints did not show significant concentration responsiveness (log-linear regression, *p* > 0.05), but did show significant effects of oil exposure by ANOVA (Fig. 4, Fig. 5, Supplemental Materials: Table S1). For Source HEWAF these endpoints included SV-YM gap and atrial contractility and *nppa* and *amhc* levels, while Source CEWAF included SV-YM gap, AV angle and atrial contractility and *nppa* and *nkx2.5* levels (Fig. 4B). For AW-Source CEWAF, these endpoints included AV angle and edema area, and *nkx2.5* levels (Fig. 4C). For AW-Source HEWAF, *nppa* response to oil exposure was not significant by ANOVA (*p* > 0.05) but did demonstrate significant concentration-responsiveness by log-linear regression (Fig. 4, Supplemental Materials: Table S1). For Slick A HEWAF, which had the lowest PAH concentrations, endpoints significant by ANOVA only included edema area, SV-YM gap, and AV angle, while Slick A CEWAF included *amhc* levels only (Fig. 5).

Thresholds for toxicity for both morphometric and molecular indicators were determined using log-linear regression against WAF dilution (e.g., Supplemental Materials: Figures S5), and based on deviation of values in oil-exposed treatments from the upper or lower 95% confidence interval of controls (e.g., Supplemental Materials: Figure S6), depending on whether a response was positive or negative, respectively. Threshold WAF dilution values were then used to calculate concentration thresholds for measured total (particulate plus dissolved) or modeled dissolved PAHs. Focusing on modeled dissolved PAH concentrations, thresholds for occurrence of edema ranged from 0.6 to 3.1 μg/L ∑PAH40 and from 0.3 to 0.9 μg/L ∑TAH. Edema occurrence was generally the indicator with the lowest threshold, although in some tests the other edema-related measures (edema area and SV-YM gap) were nearly as sensitive (Supplemental Materials: Table S1). Although AV angle and fractional shortening thresholds could not be determined for many tests, these generally were less sensitive indicators, with thresholds in the ranges of ~5 to 10 μg/L ∑PAH40, and ~2 to 3 μg/L ∑TAH, respectively (Supplemental Materials: Table S1). Notably, however, the threshold ranges for all cardiac endpoints were narrower based on ∑TAH concentrations (Supplemental Materials: Table S1).

### Rapid development of mahi mahi and time-dependency of molecular cardiotoxicity indicators

**Figure 6:**
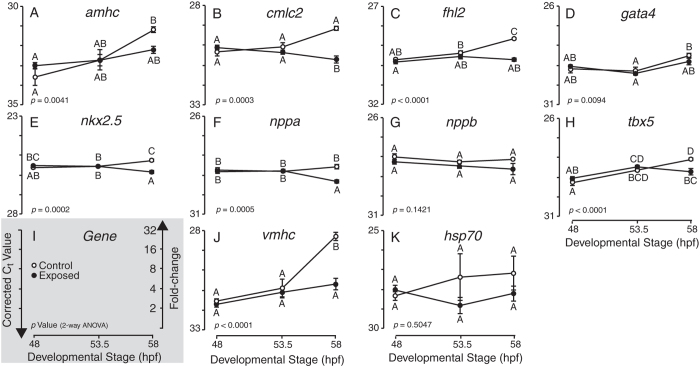
Expression of molecular cardiotoxicity indicators across developmental time in Control and AW-Source HEWAF exposed mahi mahi larvae. (**A–H,J**) Quantitative PCR (qPCR) determined transcript abundance (i.e., C_t_ values) of nine cardiotoxicity indicator genes (A, *amhc;* B, *cmlc2;* C, *fhl2;* D, *gata4;* E, *nkx2.5;* F, *nppa;* G, *nppb;* H, *tbx5;* J, *vmhc*) and *hsp70* (**K**) as a stress indicator across developmental time (48–58 hpf) in control and 0.10% AW-Source HEWAF exposed larvae. Gene-specific *p* values are from two-way ANOVA (Supplemental Materials: Table S2) and letter groupings indicate time points with statistically different expression levels (Tukey-Kramer HSD post-hoc, α = 0.05). Presented C_t_ values were corrected to geometric average of *actc1b* and *rps25* following Reference Residual Normalization^58^. (I) Legend depicting orientation of Y-axes and conversion of C_t_ to fold-change. Open symbols, control; Filled symbol, exposed. C_t_, threshold cycle. Y-axes range 5 cycles or 32-fold. Data are mean ± SEM.

The relationship between imaging/tissue collection time and sensitivity of molecular indicators (Figs 4 and 5) prompted us to compare relative transcript levels at three different developmental time points spanning the range over which samples were collected (48, 53.5, 58 hpf; see Methods). Embryos were exposed to a single concentration of AW-Source HEWAF targeted to be near the highest exposure concentration in the AW-Source HEWAF concentration-response assay above (Fig. 4C). The 0.1% dilution of HEWAF had a particulate plus dissolved ∑PAH40 concentration of 6.2 μg/L at the onset of exposures that were carried out in a modified Imhoff cone system^32^ rather than open beakers (see Methods). Consistent with the concentration-response assays, the most robust molecular indicators (two-way ANOVA, *p* < 0.05; Supplemental Materials: Table S2) were a subset of the same genes involved in contractility/hypertrophy (*cmlc2*, *fhl2*, *nppa*, *vmhc*) and also transcription factors *nkx2.5* and *tbx5* (Fig. 6). Notably however, no significant differences were observed for any molecular indicators at time points before 58 hours (i.e., 48 and 53.5 hours), which represents the latest time point sampled in the concentration-response assays with the largest number of indicators significant by the triple criteria (Source CEWAF and AW-Source CEWAF). Importantly, there was also no significant activation of a stress response in this assay, indicated by no effect on *hsp70* levels (Fig. 6, Supplemental Materials: Table S2). The gene expression profiles in control samples suggest that reduced levels of cardiac gene expression reflect a failure to up-regulate endogenous transcript abundance at 58 hpf rather than an active down-regulation of target gene transcription.

### Validation of qPCR results by *in situ* hybridization

**Figure 7:**
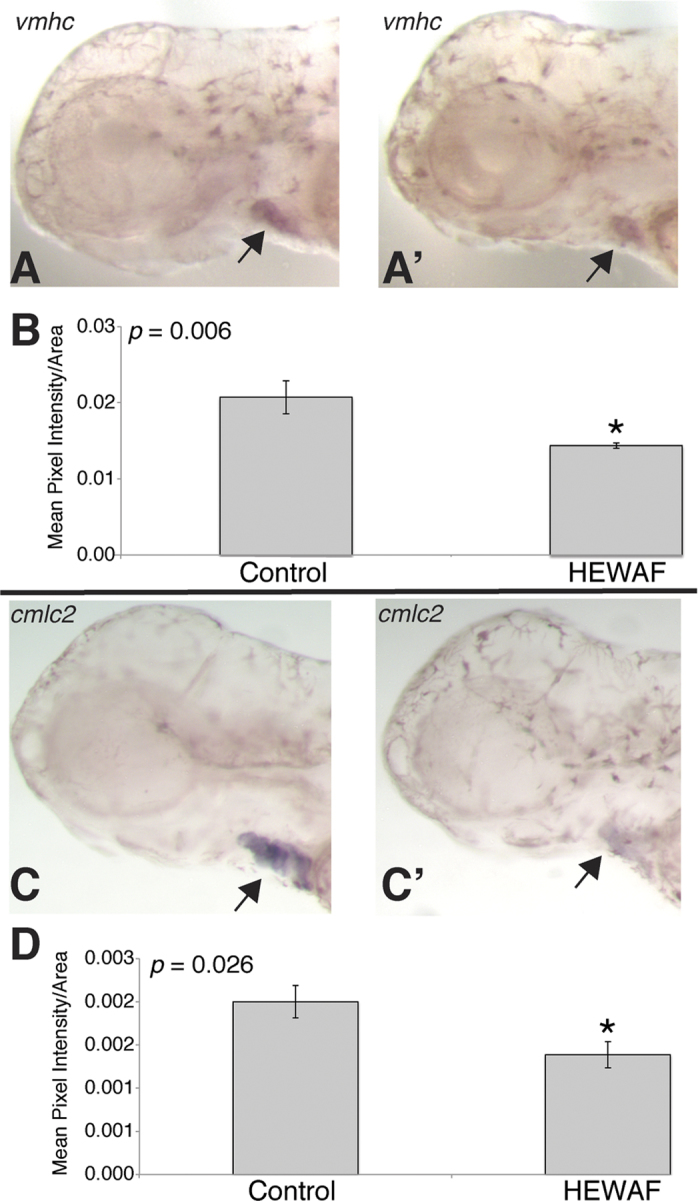
*In situ* hybridization showing endogenous localization of qPCR amplicon DIG-riboprobes in larval mahi mahi. Ventricular myosin heavy chain (*vmhc*) and cardiac myosin light chain 2 (*cmlc2*) *in situs* show ventricle- and heart-specific localization, respectively. (**A,C**) Control larvae exposed to 0.8 μg/L ΣPAH (*n* = 12). (**A′,C′**) Larvae exposed to 6.2 μg/L ΣPAH Slick A HEWAF (*n* = 13). (**B,D**) Histograms depicting area-corrected mean pixel intensity of ventricle- and cardiac-specific expression of *vmhc* (**B**) and *cmlc2* (**D**), respectively. Asterisks denote significant difference between control and HEWAF exposed (Student’s t-test, *p* < 0.05).

We used whole-mount *in situ* hybridization as a semi-quantitative method to validate qPCR results for *vmhc* and *cmlc2* using DIG-labeled riboprobes generated from the same qPCR primers and amplicons. Consistent with lower transcript abundance measured by qPCR, larvae exposed to the highest tested concentration of a Slick A HEWAF (particulate plus dissolved ∑PAH40 6.2 μg/L) show lower intensity heart labeling in whole-mount *in situ* hybridization for both *vmhc* (Fig. 7A, A’) and *cmlc2* (Fig. 7C, C’). Quantification of signal intensity by pixel analysis showed significantly lower levels of labeling for both genes (Student’s t-test, *p* < 0.05; Fig. 7B,D).

### Specificity of crude oil toxicity to the developing heart

In contrast to other pelagic species, extracardiac defects were not observed in any assay. In particular, there were no consistent abnormalities in body axis or finfolds (Fig. 2), and no craniofacial malformations or reduction in eye growth (Supplemental Materials: Figure S7A, C). There was no evidence of developmental delay, as indicated by the degree of pigmentation (Fig. 2) and the presence of normal lateral line neuromasts (Supplemental Materials: Figure S7C, D). In parallel with these morphological observations, the extracardiac gene *ikaros* was unaffected by any exposure (Supplemental Materials: Table S1). Similarly, activation of a stress response was not correlated with cardiotoxicity, as *hsp70* was up-regulated significantly (triple criteria) in only one assay (Slick A HEWAF; Supplemental Materials: Table S1) and was not affected in assays that had higher degrees of edema (e.g., Source HEWAF; Supplemental Materials: Table S1). In most tests the xenobiotic response biomarkers *cyp1a* and *cyp1b1* were concentration-dependently up-regulated as expected, with *cyp1a* being the most highly expressed gene in response to oil exposure (Supplemental Materials: Table S1). In two tests (Source and AW-Source CEWAFs), both *cyp1a* and *cyp1b1* genes were either non-responsive or a treatment effect was significant by ANOVA only. Determination of threshold concentrations for *cyp1a* induction as described above for the cardiac indicators gave a range of 0.2 to 0.5 μg/L ∑PAH40 and 0.06 to 0.3 μg/L ∑TAH (Supplemental Materials: Table S1).

## Discussion

Here we explored the extent to which developmental patterns of cardiac-specific gene expression correspond to more conventional indicators of abnormal heart development in early life stage mahi mahi, following exposure to field-collected crude oil samples from the 2010 Deepwater Horizon event. As expected from previous studies, MC252 crude oil was overtly cardiotoxic, albeit with a milder injury phenotype (i.e., without the jaw, eye, and body axis malformations that are downstream of heart failure in other fish species). Nevertheless, mahi mahi larvae displayed familiar functional and morphometric symptoms of the crude oil syndrome, which included abnormal heart chamber looping in addition to pericardial edema, and reduced heart muscle contractility. Several genes that are diagnostic of cardiovascular stress in humans were also concentration-responsive to oil exposure. Relative to visible forms of injury, these molecular indicators became relatively more sensitive as larval development progressed. Although the proximal mechanism of crude oil-derived PAH toxicity to cardiomyocytes is likely pharmacological and not transcriptional, these findings indicate that secondary changes in cardiac-specific gene expression can complement and extend traditional injury assessments in the aftermath of oil spills, particularly for fish that appear morphologically normal.

As we have previously shown, Deepwater Horizon crude oil samples were severely toxic to the embryos of other top fish predators, including bluefin and yellowfin tunas and yellowtail amberjack^9^. For these species, exposures to very low concentrations of PAHs caused a reduction in heart rate and irregular rhythm. Mahi embryos also exhibited concentration-dependent reductions in atrial contractility (ventricular contractility was not measured), but did not show evidence of craniofacial or other malformations secondary to edema. Egg size may contribute to these species-specific differences in MC252 crude oil vulnerability. Of the four pelagic species tested, mahi mahi have the largest egg diameter (1.4 mm) relative to amberjack (1.2 mm) and tunas (1 mm)^9^,^33^. The rank order of vulnerability (tunas > amberjack > mahi mahi) corresponds to respective egg surface-to-volume ratios of 6.7, 5.4, and 4.3, suggesting that smaller embryos (with larger relative surface area) accumulate higher tissue PAH concentrations more rapidly.

Subtle differences in cardiotoxicity across species may also be attributable to life history variation as it relates to cardiac function, regulation of E-C coupling, and ecophysiological characteristics (e.g., thermal tolerance). In E-C coupling, a transient rise in intracellular calcium ion concentration links electrical excitation (depolarization) of cardiomyocytes to myofiber contraction. Cardiomyocytes are returned to the resting state (repolarized) following contraction by outward movement of potassium ions (*I*_Kr_ current) through the ether-à-go-go-related gene (ERG) channel. Crude oil has a dual effect on E-C coupling, blocking both the normal cycling of intracellular calcium by blocking either the ryanodine receptor calcium channel or the sarcoplasmic reticulum calcium pump SERCA2 and the repolarizing *I*_Kr_ current^5^. Fish species vary considerably in the degree in which intracellular calcium stores (vs. extracellular) and rates of *I*_Kr_ current contribute to regulation of heart rate and contractility, reflected in some cases, for example, by the cellular levels of specific ion channels such as ERG^34^,^35^. These differences are typically related to physiological characteristics such as optimal temperature range. Similar to mahi mahi, the developing hearts of zebrafish embryos show reduced contractility in response to crude oil, with only mild bradycardia at higher exposure concentrations^3^,^10^. Our current findings suggest that heart rate in both mahi mahi and zebrafish is less dependent on *I*_Kr_, and that the predominant cardiac loss-of-function phenotype (reduced contractility) is a more prominent contribution of intracellular calcium stores to contractility, i.e., the molecular targets of PAHs in intracellular calcium handling are more abundant or sensitive than the potassium channels regulating *I*_Kr_ in these species. This may reflect a narrower thermal tolerance range for mahi mahi and zebrafish relative to deeper diving tunas^36^,^37^,^38^.

The establishment of a captive broodstock of mahi mahi that have been volitionally spawning routinely at the University of Miami Experimental Hatchery (UMEH) facilitated a broader range of tests than those conducted previously with tunas^9^. It was nevertheless challenging to phenotypically anchor patterns of gene expression changes to visible cardiac injury in crude oil-exposed fish. In addition to displaying a mild crude oil toxicity syndrome, mahi mahi develop much more rapidly than focal species for past oil spill impact assessments (e.g., salmon, herring), and this contributed to background variation in gene expression during the period of digital data collection. Despite this ontogenetic variability, the morphological and molecular indicators proved robust for PAH exposures at the low end of the concentration-response relationship (generally below 10 μg/L), with one or more significantly responsive indicators for every assay. Given the limitations stemming from the inability to culture these pelagic embryos at higher densities, the robustness of all indicators would most likely only improve with assays using a larger number of individuals. As was evident from the paired HEWAF/CEWAF tests using Source and AW-Source oils, morphometric and molecular indicators were more robust at earlier and later developmental time points, respectively. The declining robustness of visible effects may reflect a toxicokinetic loss of PAHs over time (metabolism and depuration), with some recovery of cardiac function during larval development in clean seawater. For example, zebrafish embryos exposed transiently to crude oil recover cardiac function and circulation when they hatch in clean water^4^,^10^. The increasing robustness of molecular indicators over developmental time is consistent with a harmful shift away from normal cardiac morphogenesis, despite a recovery of function. This has been demonstrated from anatomical assessments of heart structure at later life stages in other species^17^,^18^.

Given that crude oils are complex chemical mixtures, developmental toxicity is likely to arise from multiple mechanisms. For cardiac phenotypes, ion channel blockade and the disruption of E-C coupling among individual cardiomyocytes likely plays a central role^3^,^5^. However, there is some evidence for an additional involvement of the aryl hydrocarbon receptor (AHR) pathway^8^,^11^,^39^. AHR is the ligand-dependent transcriptional activator that controls the battery of genes that function in PAH metabolism^40^, including *cyp1a* and *cyp1b1*. Similar to potent but poorly metabolized AHR ligands like dioxins and PCBs^41^, some PAHs (i.e., primarily higher molecular weight compounds that are absent from dissolved petrogenic mixtures) cause cardiotoxicity mediated by inappropriate AHR activation in the developing heart^22^,^42^,^43^. It is also possible that some alkylated tricyclic PAHs that are abundant in crude oil (e.g., C3-phenanthrenes) drive toxic cardiomyocyte AHR activation^11^,^39^. Although the connections between AHR transcriptional activation and the down-regulation of cardiac genes corresponding to embryo/larval heart failure have not been identified, AHR-driven cardiotoxicity is clearly mediated by transcription. Indeed, down-regulation of genes involved in cardiac contractility is a relatively rapid response to AHR-dependent toxicants such as the 5-ring PAH benzo(a)pyrene^44^. At the same time, there is little evidence that transcriptional regulation plays a major role in the homeostatic maintenance of E-C coupling, which instead may be controlled largely by post-transcriptional and post-translational mechanisms^45^.

Our selection of candidate genes for the study was hypothesis-driven, from comparisons between crude oil cardiotoxicity phenotypes and known loss-of-function mutations in zebrafish, as well as markers for human heart failure and pathological hypertrophy that have conserved responses in zebrafish. The natriuretic peptide genes *nppa* and *nppb* were anticipated to be up-regulated, as they are in human heart failure^26^ and many zebrafish cardiac function mutants with reduced contractility (e.g., ref. 46). Rather, we found that *nppa*/*nppb* were down-regulated, suggesting that in mahi mahi the disruption of intracellular calcium cycling by crude oil interferes with the mechanotransduction process by which vascular tone regulates *nppa*/*nppb* transcription, possibly through links between integrin-linked kinase and the sarcoplasmic reticulum Ca^2+^ pump (SERCA2)^47^,^48^. Nevertheless, reduced expression of natriuretic peptide genes, other structural and regulatory components of the contractile apparatus (*amhc*, *cmlc2*, *vmhc*), and *fhl2* are all consistent with a persistent loss-of-function phenotype. Given the normal roles for *cmlc2* and *vmhc*, changes in the expression of these genes could signal a pathological ventricular response to an earlier reduction in atrial fractional shortening^46^. Reduced levels of *tbx5* are consistent with functional defects related to calcium cycling^49^ as well as a shift in heart development towards cardiomyopathy pathways^50^. Compared to the more rapid responses downstream of AHR signaling in the developing fish heart^44^, the delay in detectable changes in these crude oil molecular indicators suggests a minimal direct role, if any, for chronic AHR activation by PAHs.

In summary, microscopically visible measures of cardiac injury were more diagnostic of MC252 crude oil toxicity at earlier developmental time points in mahi mahi. However, as larval development progressed changes in cardiac-related gene expression became more sensitive. Past studies with species more amenable to detailed microscopy at the embryonic stage (i.e., relative to hatched larvae) have shown that crude oil causes defects in cardiac function soon after a regular heartbeat is established, and well before hatch^3^,^51^. While we have not identified molecular indicators for these early functional defects in response to a disrupted E-C coupling, the genes profiled here may be diagnostic of long-term impacts to the cardiovascular systems of fish that survive embryonic crude oil exposures (e.g., altered heart shape), as a basis for a permanent loss of physiological capacity and a corresponding increase in delayed mortality^15^,^17^,^18^. Our findings also suggest that it may be necessary to tailor molecular indicators of crude oil cardiotoxicity to individual species. Although crude oils appear to be universally toxic to developing fish hearts, species-specific variation in cardiac physiology or toxicokinetics are likely to dictate the precise etiology of the syndrome at a molecular level.

## Methods

### Animals

Wild mahi mahi were captured by hook and line off the coast of Miami, FL and then transported to the University of Miami Experimental Hatchery (UMEH). The captive broodstock was maintained in 80 m^3^ fiberglass maturation tanks equipped with re-circulated and temperature controlled water (IACUC protocol # 12-064). Embryos were collected within 2–10 hr following volitional (i.e., non-induced) spawn using standard UMEH methods^52^. The embryos were treated with a formalin prophylactic (37% formaldehyde solution at 100 ppm for 1 hr) and then rinsed for 30 min, where a minimum of 300% water volume in the treatment vessel was exchanged using filtered, UV-sterilized seawater. A small sample of eggs was collected from each spawning event to microscopically assess fertilization rate and embryo quality. Spawns demonstrating low fertilization rate (<85%) or frequent developmental abnormalities (>5%) were not used.

### Water-accommodated fraction (WAF) preparation and PAH analysis

All oil samples used in toxicity assays were collected under chain of custody during Deepwater Horizon disaster response efforts. These included source oil (Source) collected from the damaged wellhead’s riser pipe (sample 072610–03), source oil that was artificially weathered (AW-Source) by heating with gentle mixing to 90–105 °C until the mass was reduced by 33–38% (sample 072610-W-A), and an ocean surface sample (CTC02404-02) collected 29 July 2010 from a barge holding mixed oil offloaded from a number of different skimmers (Slick A). High-energy WAFs (HEWAFs) were prepared using a commercial stainless steel blender as described elsewhere^10^. Chemically enhanced WAFs (CEWAFs) were prepared with the dispersant COREXIT 9500 using a standardized low-energy method^53^. WAF stocks and associated dilutions for mahi mahi early life stage exposures were constituted in filtered seawater. WAF nominal dilutions were validated by a fluorescent method, and PAHs analyzed by gas chromatography/mass spectrometry both detailed elsewhere^25^. A total of 50 individual PAHs were measured^25^. For comparison to previous work on other pelagic species^9^, “∑PAH” values here refer to the sum of 40 PAH analytes indicted in Fig. 1, while the sum of 50 analytes is indicated by “∑PAH50”. The additional analytes included in the ∑PAH50 are additional alkylated fluoranthenes, parent and alkyl-naphthobenzothiopenes, and benzo(a)fluoranthene. These were minor contributors to the total, representing a maximal difference of about 6% between ∑PAH50 and ∑PAH40 in weathered oils, and less in unweathered source oil. Moreover, water chemistry analysis of the 93 samples that encompass the three oil types (Slick A, AW-Source, Source) and two WAF preparation methods (HEWAF and CEWAF) presented herein yielded a mean ∑PAH50:∑PAH40 ratio of 1.064 ± 0.044.

### Embryonic exposures

As described in detail elsewhere^25^, crude oil exposures were performed in a temperature controlled environmental chamber (26 °C) with a 16:8 light/dark cycle. Treatments were generated by spiking 5 L of 1 μm filtered, UV-sterilized seawater with varying levels of WAF using a glass syringe. The treatment was mixed on a stir plate for 5 min and then aliquoted into four 1-L replicates. A single replicate consisted of 20 embryos in 1-L of test solution held in a 1-L glass beaker, with four replicates per treatment. Exposures were continuous for 48 hr without water exchange, and tests were not included if control survival was less than 70% at hatching.

An additional set of exposures was performed to assess the time-dependency of molecular cardiotoxicity indicators. Briefly, these exposures were performed in custom-built pelagic embryo-larval exposure chambers, each of which consists of a 1 L glass Imhoff cone customized with an overflow spout for draining into a 1 liter glass beaker and a Teflon stopcock on the bottom^32^. Total test solution volume was 1.8 L and embryos/larvae were retained in the cone using a glass excluder extending from the overflow drain with nylon mesh fastened on both sides with silicone o-rings. Relative to exposures in beakers, this paradigm likely yielded PAH concentrations that declined more quickly due to increased surface area and recirculation.

### Digital imaging

Mahi mahi larvae were captured from exposure vessels without the use of anesthetic (2–3 at a time), transferred to a petri dish, and then mounted individually atop 2% methylcellulose in seawater. Two stereoscope stations allowed parallel processing, and larvae were imaged continuously until all replicates were completed. Digital images of oil-exposed fish were collected in random treatment order, with control fish spaced evenly throughout the sample-processing interval (5–8 hrs). Images and video (640 × 480 pixels) were acquired using FireI-400 industrial digital video cameras (Unibrain, San Ramon, Ben Software, CA) mounted on Nikon SMZ800 stereomicroscopes, using MacBook laptops (Apple, Cupertino, CA) and either BTV Carbon Pro or iMovie software. A stage micrometer provided calibration.

### Morphometric analyses

For scoring of presence or absence of edema, still frames and videos were assessed for the shape of the yolk mass. Larvae were scored as normal if the anterior portion of the yolk sac was smooth and rounded with a bullet-shaped tip and if there were no obvious indentations on the yolk sac due to pressure from fluid buildup in the pericardial area. Edema was scored positive if the anterior portion of the yolk sac was concave or pushed to a sharp point, and/or if indentations indicated by dark, angular lines were seen pushing on the yolk sac due to pressure from fluid buildup in the pericardial area. There was a range of normal yolk sac shapes in control fish. Sometimes the yolk sac did not have a perfect rounded, bullet shape (e.g., blunted or semi-pointed), but the larva was still considered within the range of normal.

Measurements of edema area, sinus venosus-yolk mass gap (SV-YM) and atrioventricular (AV) angle were made using ImageJ software (rsbweb.nih.gov/ij/). For measurements of edema area a line was drawn enclosing the pericardial area plus any portion of the yolk mass distorted by accumulation of fluid using the ImageJ freehand tool. In the absence of visible edema this line typically enclosed the area anterior to the yolk sac and beneath the jaw cartilage, and sometimes extended along a thin space underneath the yolk mass. In larvae with edema, the line enclosed the fluid-filled area pushing against the yolk mass. To be consistent, lines were drawn within the boundaries of the yolk sac only if a sharp, angular, and defined distortion of the yolk mass was evident. An independent measure of fluid accumulation (SV-YM gap) was determined from video files opened in ImageJ and paused at a frame where the connection of posterior end of the atrium to the presumptive sinus venosus was visible. Using the ImageJ line tool, a line was drawn on the same axis as the center of the atrium, parallel to the atrial walls, starting from the posterior end of the atrium to the yolk mass. For AV angle, videos were opened in ImageJ and stopped at a frame in which the atrium was maximally relaxed and the ventricle maximally contracted. At this point the line tool was used to draw a line through the center of the ventricle starting at the anterior end and ending at a point that lined up visually with the center of the posterior opening of the atrium, with a second line segment created from that point to the posterior atrial opening. The angle between the two line segments was then measured. Three measurements were taken for each animal, with the median value used for statistical analysis.

Cardiac function was assessed in ≥10-sec digital video clips of all viable larvae collected from each replicate exposure. Fractional shortening (FS) was determined by measuring the atrial diameter during systole and diastole with ImageJ, and calculated with the following formula:

(Diastolic diameter – systolic diameter)/Diastolic diameter  ×  100

### Candidate Gene Identification and Primer Design

A total of 10 putative cardiac-specific (alpha, actin, cardiac muscle 1b, *actc1b*; atrial myosin heavy chain, *amhc*; cardiac myosin light chain 2, *cmlc2;* four and a half LIM domains 2, *fhl2;* GATA4 binding protein 4, *gata4*; NK2 homeobox 5, *nkx2.5;* natriuretic peptide A, *nppa*; natriuretic peptide B, *nppb*; T-box 5, *tbx5*; ventricular myosin heavy chain, *vmhc*) and 5 extracardiac (cytochrome P4501a, *cyp1a*; cytochrome P4501b1, *cyp1b1*; heat shock protein 70 kDa, *hsp70*; Ikaros, *ikaros*; 40 S ribosomal protein sub-unit 25, *rps25*) molecular indicators were identified as candidate genes from the fish and human cardiotoxicity literature (Table 2). For each putative molecular indicator, National Center for Biotechnology Information (NCBI) nucleotide database was mined for available Perciform sequences (e.g., *Coryphaena hippurus, Lates calcarifer, Haplochromis burtoni, Dicentrarchus labrax, Sparus aurata, Pundamilia nyererei, Maylandia zebra, Neolamprologus brichardi*, *Oreochromis niloticus*, etc.) and available sequences were aligned with ClustalW (http://www.genome.jp/tools/clustalw/). Identified regions of conservation were then targeted for qPCR primer design with Primer3^54^, except *amhc* as primers were designed using available mahi mahi-specific sequence ([JX190488]^55^). Designed primers were target verified (≥80% sequence identity) by NCBI Primer-BLAST^56^ and amplicons were sequenced and target verified by nucleotide BLAST (Supplemental Materials: Table S3).

### RNA Extraction, cDNA Synthesis, and Quantitative PCR

Total RNA was extracted from pools of snap-frozen larvae by mechanical homogenization (T8 Ultra Turrax homogenizer with S8N-5G dispersion element; IKA Labortechnik, Staufen, Germany) in TRIzol™ Reagent (Invitrogen Inc., Irvine, CA) and subsequently purified and DNAse-treated with DirectZol™ spin-columns (Zymo Inc.), following manufacturer’s instructions. First strand cDNA was synthesized from a normalized quantity of total RNA using SuperScriptIII™ Reverse Transcriptase with dT_20_ primer (Invitrogen Inc.; Invitrogen and Applied Biosystems have been merged into Life Tech and now ThermoFisher, Grand Island, NY), following manufacturer’s instructions. Quantitative PCR (qPCR) reactions were run in duplicate using SYBR Green™ chemistry (Applied Biosystems Inc.), 250 nM each primer, and 4 ng template on a Viia7™ Real-Time qPCR Detection System (Applied Biosystems Inc.) for 40 cycles under standard cycling conditions (60°C annealing) in optical 96-well plates (Applied Biosystems Inc.). Dissociation curves were generated as terminal step of all qPCR reactions to verify single product amplification. All primer pairs demonstrated acceptable efficiency (90–105%^57^) and relative expression data for each gene was normalized to the geometric average of two technical reference genes, one cardiac-specific (*actc1b*) and one ubiquitous ribosomal protein subunit (*rps25*), as detailed elsewhere^58^.

### Whole-mount *in situ* hybridization

DNA template for sense and anti-sense RNA probes (i.e., riboprobes) were generated by end-point PCR using qPCR primers with appended universal T7 and SP6 promoter sequences^59^. DIG-labeled riboprobes were subsequently synthesized *in vitro* using the DIG RNA Labeling Kit (Roche Inc., Sigma-Aldrich, St. Louis, MO), DirectZol™ column purified, and diluted to [1 ng/μL] working stock concentration in Hyb^+^ buffer (50% formamide, 5× sodium sulfanyl citrate (SSC), 50 μg/ml Heparin, 500 μg/ml yeast transfer RNA, 0.1% Tween-20, and citric acid to pH 6.0). Whole mount *in situ* hybridization^59^ included the following modifications: 1) proteinase K treatment reduced to 5 min and 4% paraformaldehyde refixation increased to 30 min; 2) pre-hybridization, hybridization and post-hybridization washes conducted at 61 °C; 3) pre-hybridization in Hyb^+^ prolonged to overnight. Hybridization was terminated with stop buffer (1 mM EDTA in phosphate buffer solution, pH 5.5) when heart staining was clearly visible. Stained larvae were mounted in 2% methylcellulose and digital images were acquired using the SPOT imaging system (Diagnostic Instruments, Sterling Heights, MI; camera: model 2.3.1; software: version 4.5.9.9) mounted on a Nikon SMZ800 stereomicroscope. Lighting, exposure and magnification were held constant across all repeat images. Pixel intensity measurements of heart-specific staining were made using ImageJ software (rsbweb.nih.gov/ij/) by drawing a line that encircled the stained heart using the freehand tool and subsequently corrected to the average area of encircled heart staining, which did not differ between control and exposed fish for either *vmhc* or *cmlc2* (Student’s t-test, *p* > 0.05).

### Statistical analyses

Statistical analyses and generation of plots were carried out with JMP10 (SAS Institute, Cary, NC), Prism 5 (GraphPad Software, La Jolla, CA), and KaleidaGraph 4.5 (Synergy, Reading, PA) for Macintosh. Gene expression and morphometric data were assessed for treatment effect using one-way analysis of variance (ANOVA) with Tukey-Kramer HSD post-hoc analysis as well as linear regressions on log_10_-transformed %WAF values (Supplemental Materials: Table S1). ANOVAs and log-linear regressions of gene expression data were conducted on log_2_-transformed fold-change values^58^. Control data (0% WAF exposure) were not included in the regression; rather, the intersection of the regression with the upper or lower bounds of the 95% confidence interval for the controls was used to determine the response threshold (Supplemental Materials: Figure S5). For all metrics that exhibited significant non-zero regression slopes (i.e., concentration-responsive), threshold %WAF values were calculated using the following equation:


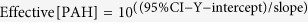


where CI is the lower or upper 95% confidence interval of the mean of the control treatment if the slope of the regression is negative or positive, respectively (Supplemental Materials: Figure S5). Threshold %WAF values for each exposure were only considered relevant if within the range empirically tested by that experiment (Supplemental Materials: Figure S6, Table S1). All gene expression and morphometric data are plotted using %WAF (i.e., common unit) to prevent *a priori* assumptions regarding major cardiotoxic constituents in HEWAF/CEWAF complex mixtures (Figs 4 and 5, Supplemental Materials: Figure S5). Moreover, performing analyses using %WAF common unit permits subsequent extrapolation(s) to constituent PAHs of interest for each WAF tested (Supplemental Materials: Figure S6). Expression of each molecular indicator in control and 0.1% AW-Source HEWAF exposed larval mahi mahi over developmental time (Fig. 6, Supplemental Materials: Table S2) was analyzed using discrete two-way ANOVAs with Age, Treatment and Age*Treatment as factors and Tukey-Kramer HSD post-hoc analyses. Significant differences in area corrected pixel intensity of *vmhc* and *cmlc2 in situ* hybridizations (Fig. 7B,D) were determined by Student’s t-test. For all statistical comparisons, means were considered significantly different when *p* < 0.05.

## Additional Information

**GenBank Accession Codes**: JX190488 (Article). JX190488, NM_001145995, DQ225183, NM_001279627, NM_001104912, AB162777, AB162778, NM_001201500, NM_001112733, EU163982, AJ249074, AB436470, GQ466344, NM_131591, AB291578 (Supplemental Materials: Table S3).

**How to cite this article**: Edmunds, R. C. *et al.* Corresponding morphological and molecular indicators of crude oil toxicity to the developing hearts of mahi mahi. *Sci. Rep.* **5**, 17326; doi: 10.1038/srep17326 (2015).

## Supplementary Material

Supplementary Information

## Figures and Tables

**Table 1:** Summary of WAF dilutions and PAH concentrations for mahi mahi exposures.

Preperation^2^	WAF Dilutions (%)					ΣPAH50 (μg/L)					ΣPAH40 (μg/L)					ΣTAH (μg/L)					dΣPAH50 (μg/L)					dΣPAH40 (μg/L)					dΣTAH (μg/L)				
S-HEWAF	0.0	0.0	0.1	0.1	0.3	0.1	3.5	6.4	10.3	21.6	0.1	3.4	6.0	9.8	20.3	0.0	1.2	2.2	3.2	6.8	0.1	3.0	5.1	8.0	15.2	0.1	2.9	5.0	7.9	15.1	0.0	0.9	1.6	2.2	3.8
S-CEWAF	0.0	0.1	0.3	0.6	1.4	0.1	1.3	2.4	4.0	8.5	0.1	1.2	2.4	4.0	8.5	0.0	0.2	0.4	0.7	1.6	0.1	1.1	2.1	3.5	7.3	0.1	1.1	2.1	3.5	7.3	0.0	0.1	0.2	0.4	0.9
AW-HEWAF	0.0	0.0	0.0	0.1	0.2	0.2	2.4	4.1	7.9	15.9	0.2	2.2	3.9	7.3	14.6	0.1	1.0	1.6	3.3	6.2	0.1	1.1	2.0	3.6	6.9	0.1	1.1	1.9	3.5	6.8	0.0	0.4	0.7	1.4	2.5
AW-CEWAF	0.0	0.0	0.1	0.1	0.3	0.2	1.5	2.7	5.4	10.9	0.2	1.4	2.5	5.0	10.5	0.1	0.6	1.1	2.2	4.4	0.1	0.7	1.3	2.4	5.1	0.1	0.7	1.3	2.4	5.0	0.0	0.2	0.4	0.7	1.4
SA-HEWAF	0.0	0.0	0.0	0.1	0.1	0.2	0.4	1.2	4.8	5.1	0.2	0.4	1.1	2.2	5.1	0.0	0.1	0.5	1.3	2.7	0.2	0.3	0.6	2.6	2.6	0.2	0.3	0.6	1.2	2.6	0.0	0.1	0.3	0.7	1.4
SA-CEWAF	0.0	0.1	0.2	2.2	9.1	ND					ND					ND					ND					ND					ND				

Oil samples are ordered from least (Source) to most (Slick A) weathered. S, Source oil; AW, Artificially Weathered oil; SA, Slick A oil; HEWAF, high-energy water accommodated fraction; CEWAF, chemically-enhanced water accommodated fraction; ΣPAH50, measured sum of 50 PAHs (particulate plus dissolved); ΣPAH40, measured sum of 40 PAHs (particulate plus dissolved); ΣTAH, measured sum of tricyclic PAHs (particulate plus dissolved); dΣPAH, modeled sum of dissolved total PAHs; dΣTAH, modeled sum of dissolved tricyclic PAHs. *interpolated values derived from known higher and lower dilutions. ND, total and dissolved fractions not determinable.

**Table 2:** Molecular indicators quantified following HEWAF and CEWAF oil exposures.

Gene	Indicator Role	Function	Tissue
*amhc*	cardiotoxicity	contractility - structural	cardiac (atrium)
*cmlc2*	cardiotoxicity	contractility - regulatory	cardiac
*fhl2*	cardiotoxicity	unknown/hypertrophy	cardiac
*gata4*	cardiotoxicity	cardiogenesis - transcription	cardiac
*nkx2.5*	cardiotoxicity	cardiogenesis - transcription	cardiac
*nppa*	cardiotoxicity	contractility - regulatory	cardiac
*nppb*	cardiotoxicity	contractility - regulatory	cardiac
*tbx5*	cardiotoxicity	cardiogenesis - transcription	cardiac, pectoral fin bud
*vmhc*	cardiotoxicity	contractility - structural	cardiac (ventricle)
*ikaros*	general development	lymphopoesis - transcription	hematopoietic
*hsp70*	stress response	chaperone	ubiquitous
*cyp1a*	xenobiotic response	PAH metabolism	ubiquitous
*cyp1b1*	xenobiotic response	PAH metabolism	ubiquitous
*actc1*	intracardiac reference	contractility	cardiac
*rps25*	ubiquitous reference	ribosomal protein	ubiquitous
