# Supplementary material for: Corresponding morphological and molecular indicators of crude oil toxicity to the developing hearts of mahi mahi
Source: Sci Rep. 2015 Dec 10;5:17326. doi: 10.1038/srep17326 (PMC4674699; doi:10.1038/srep17326)
Supplement: Supplementary Information [file srep17326-s1.pdf]

Corresponding morphological and molecular indicators of crude oil toxicity to the developing hearts of mahi mahi

Richard C. Edmunds[1], J. A. Gill[2], David H. Baldwin[3], Tiffany L. Linbo[3], Barbara L. French[3], Tanya L. Brown[2], Andrew J. Esbaugh[4], Edward M. Mager[5], John Stieglitz[5], Ron Hoenig[5], Daniel Benetti[5], Martin Grosell[5], Nathaniel L. Scholz[3], and John P. Incardona[3]

[1]National Research Council Associate Program, under contract to Northwest Fisheries Science Center, National Marine Fisheries Service, NOAA, 2725 Montlake Blvd. E., Seattle, WA 98112 USA

[2]Frank Orth and Associates, under contract to Northwest Fisheries Science Center, National Marine Fisheries Service, NOAA, 2725 Montlake Blvd. E., Seattle, WA 98112 USA

[3]Environmental and Fisheries Science Division, Northwest Fisheries Science Center, National Marine Fisheries Service, NOAA, 2725 Montlake Blvd. E., Seattle, WA 98112 USA

[4]Department of Marine Science, University of Texas, Marine Science Institute, 750 Channel View Dr., Port Aransas, TX 78373 USA

[5]Department of Marine Biology and Ecology, University of Miami, Rosenstiel School of Marine and Atmospheric Science, 4600 Rickenbacker Cswy., Miami, FL 33149 USA

Supplementary Information

Figures S1 – S7
Tables S1 – S3

Figure S1

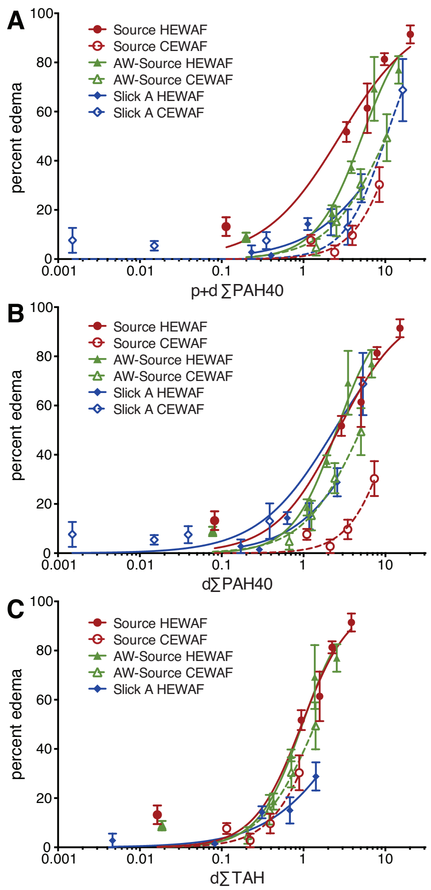

Figure S1. Incidence of edema was generally dependent on PAH concentrations. Relationship between percent edema and (A) particulates plus dissolved sum of 40 polycyclic aromatic hydrocarbons (PAHs; p+d∑PAH40), (B) dissolved sum of 40 PAHs (d∑PAH40), and (C) dissolved sum of 40 tricyclic aromatic hydrocarbons (TAHs; d∑TAH40). All measures were made in digital video frames (see Methods) and plotted with lines representing non-linear models. As described in the text, these regressions were used for graphical representation only and not statistical analyses (e.g., Figures 4, 5, Supplemental Materials: Table S1). p+d∑PAH40, actual measured values in WAF dilutions; d∑PAH40, and d∑TAH40 represent modeled dissolved concentrations.

Figure S2

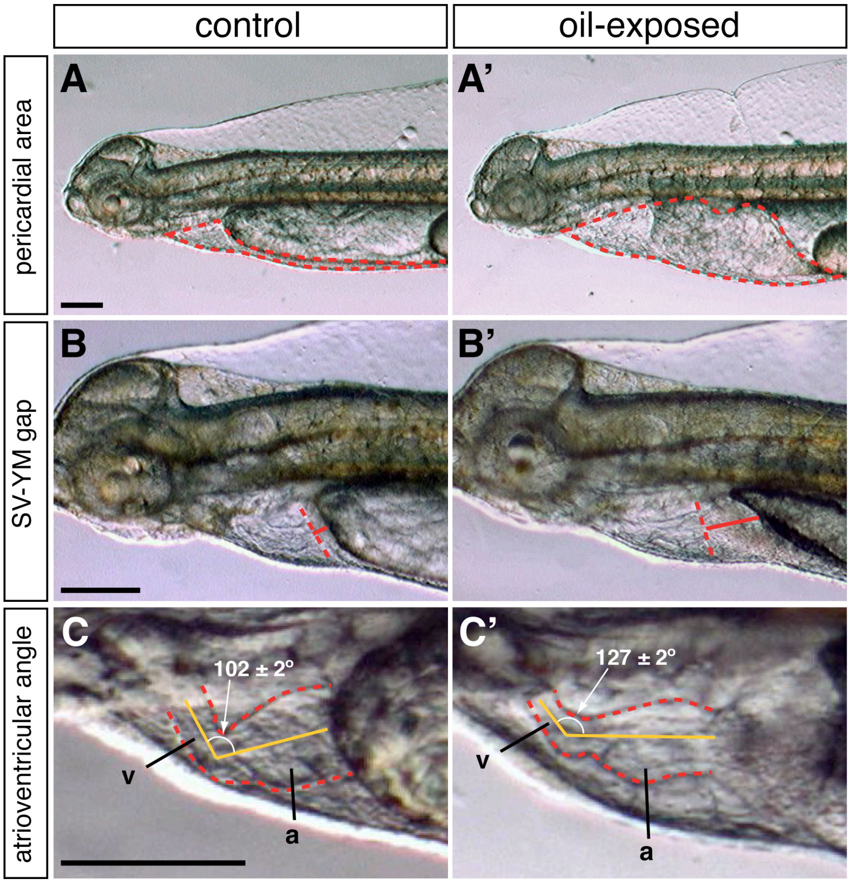

Figure S2. Morphometric analyses of cardiac phenotypes in larval mahi mahi. Representative frames from digital videos provide examples of morphological measurements made in ImageJ (see Methods). (A, A') Edema Area. Dashed red line indicates area outlined on each image and considered edema area in control (A) and oil-exposed (A') larvae. (B, B') Sinus venosus-yolk mass gap. Solid red line indicates distance between the sinus venosus (dashed red line) and the yolk mass in control (B) and oil-exposed (B') larvae. (C, C') Atrioventricular angle. Dashed red line indicates outline of heart tube and yellow line indicates the midline of each chamber, with the atrioventricular angle (± SEM) measured as the angular shift between the ventricular and atrial midlines in control (C) and oil-exposed (C') larvae. v ventricle; a, atrium. Scale bar is 1 mm.

Figure S3

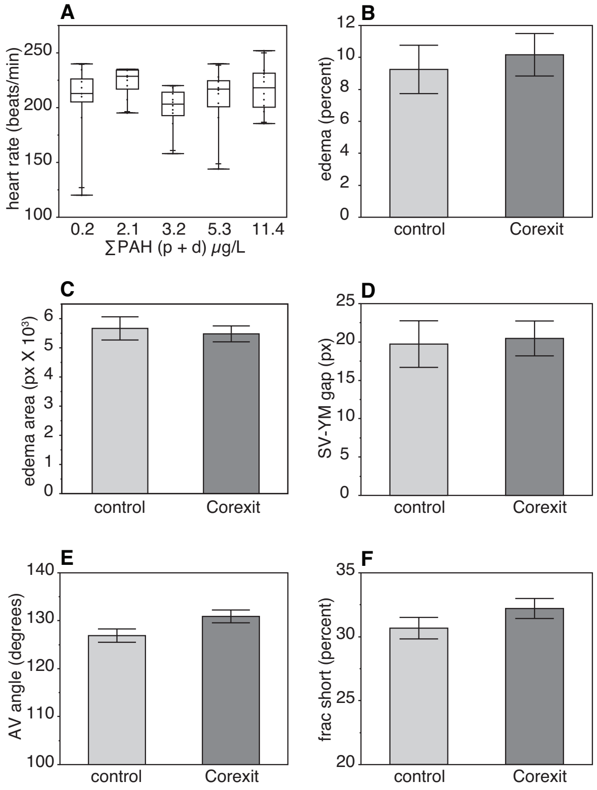

Figure S3. Lack of effects of MC252 oil exposure on larval heart rate and Corexit exposure on morphological cardiotoxicity indiators. (A) Heart rate (beats per min) calculated from digital videos for 10 fish per treatment following Source HEWAF exposure. Measured ∑PAH40 included particulate (p) plus dissolved (d) fractions in control and four HEWAF. Box-and-whisker plots encompass all data points, with mean lines indicated within the boxes (B – F) Morphological measures of cardiotoxicity endpoints in larvae exposed to 10% Corexit 9500 were obtained from digital videos as described in Methods. (B) Incidence of edema. (C) Edema area. (D) Sinus venosus-yolk mass (SV-YM) gap. (E) Atrioventricular (AV) angle. (F) Atrial contractility measured as fractional shortening (frac short) in control and Corexit exposed larval mahi mahi. Data are mean ± SEM for four replicate groups.

Figure S4

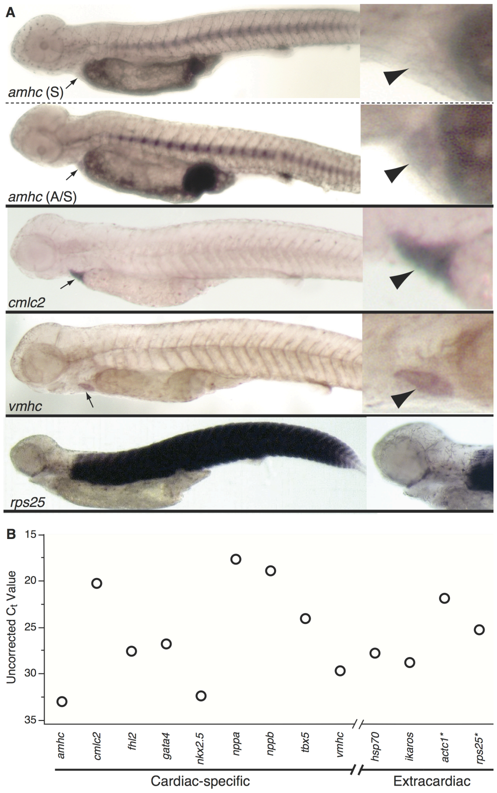

Figure S4. Validation of endogenous localization of qPCR amplicons in whole larvae and adult ventricle by *in situ* hybridization and qPCR, respectively. (A) Whole-mount *in situ* hybridization of anti-sense (*A/S*) riboprobes for three cardiac-specific molecular indicators (*amhc, cmlc2, vmhc*) and one ubiquitous 40S ribosomal protein sub-unit reference (*rps25*) (see Methods, Table 2). Sense control (*S*) is shown for *amhc* given non-specific staining in spine/neural tube. Insets show localization of cardiac-specific expression, except for reference gene *rps25*, which was not predicted to localize in larval cardiac tissue. (B) Comparison of qPCR threshold cycle (C_t) values (i.e., transcript abundance) for molecular cardiotoxicity indicators in adult ventricle tissue. Note that Y-axis is inverted (i.e., transcript abundance increases as Y-axis value decreases). Asterisks denote reference genes used for normalization.

Figure S5

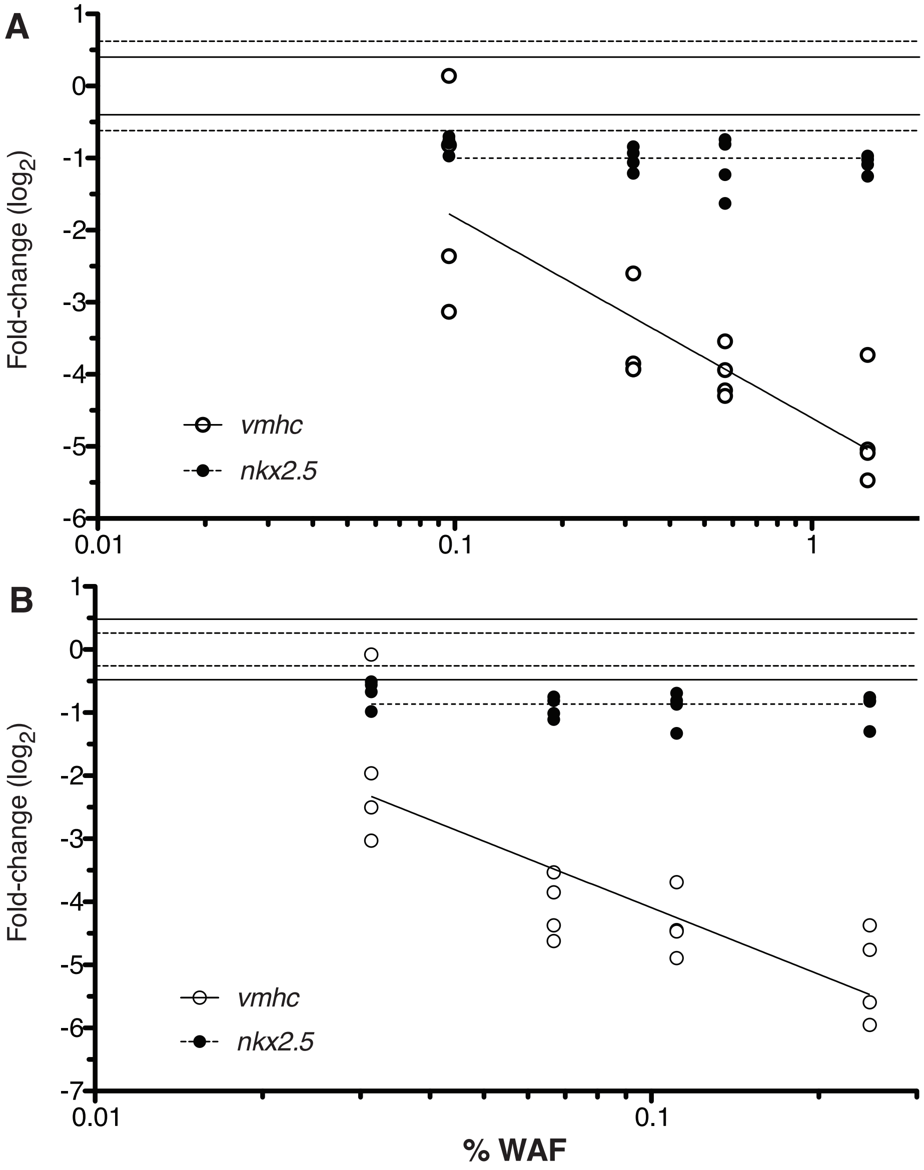

Figure S5. Log-linear relationship between WAF dilutions and log$_2$-transformed fold-change in gene expression. Ventricular myosin heavy chain (*vmhc*) and NK2 homeobox 5 (*nkx2.5*) represent molecular indicators that exhibit triple and single statistical criteria, respectively (see Results). Solid and dashed lines represent *vmhc* and *nkx2.5* log-linear regression lines of exposed fish and 95% confidence intervals of control means, respectively. (A) Source CEWAF, (B) AW-Source CEWAF.

Figure S6

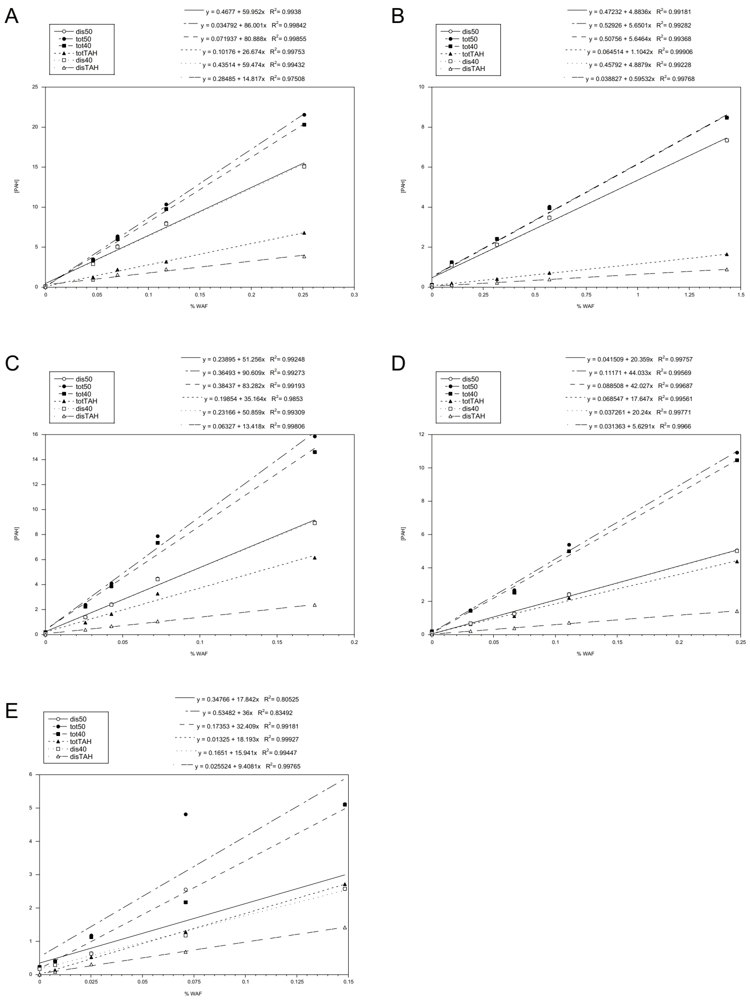

Figure S6. Linear regression relationships between WAF dilutions and constituent PAH concentrations. Linear relationship between WAF dilutions and constituent PAHs (Figure 3) for (A) Source HEWAF, (B) Source CEWAF, (C) AW-Source HEWAF, (D) AW-Source CEWAF, (E) Slick A HEWAF. These strong relationships ($p < 0.01$; $r^2 > 0.95$) permit confident extrapolation (Table 1, Supplemental Materials: Table S1). ΣPAH40, sum of measured 40 PAHs; ΣTAH, sum of measured tricyclic PAHs; dΣPAH, sum of modeled dissolved total PAHs; dΣTAH, sum of modeled dissolved tricyclic PAHs.

Figure S7

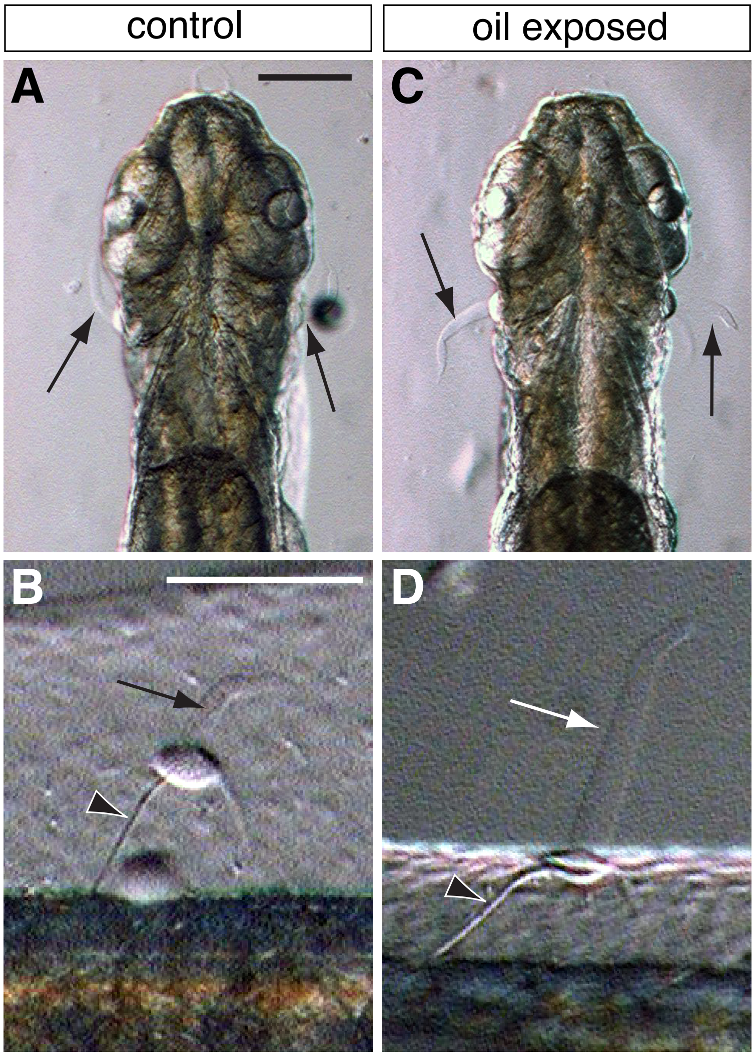

Figure S7: Normal craniofacial and lateral line development in oil-exposed mahi mahi. Ventral view of the head (A, C) and higher magnification view of the L1 neuromast (B, D) in control (A, B) and oil-exposed (C, D) mahi mahi larvae. Representative larvae are shown for control (A) and the highest exposure level (C; 14.6 μg/L ∑PAH40) from the AW-Source HEWAF exposure, and control (B) and highest exposure level (D; 16.4 μg/L ΣPAH) from the Slick A CEWAF exposure. Arrows indicate stereocilia of anterior neuromasts (A, C) or L1 posterior neuromasts (B, D); arrowheads indicate sensory neuron projections from the L1 neuromasts.

Table S1. Summary of analyses conducted on molecular and morphometric indicators.

Source HEWAF

	ANOVA	Regression				[PAH] Extrapolation					
Cardiotoxicity	*p value*	Slope	r2	*p value*	%WAF	∑PAH50	d∑PAH50	∑PAH40	d∑PAH40	∑TAH	d∑TAH
amhc	0.0364	0.009	0.0000	0.9853	NS						
cmlc2	0.1476	0.240	0.0794	0.2905	NS						
fhl2	0.1890	-0.068	0.0029	0.8431	NS						
gata4	0.1973	0.726	0.1074	0.2154	NS						
nkx2.5	0.1063	0.740	0.1179	0.1929	NS						
nppa	0.0279	-0.303	0.1720	0.1102	NS						
nppb	0.2036	-0.260	0.0747	0.3243	NS						
tbx5	0.1120	0.833	0.1228	0.1832	NS						
vmhc	0.2539	0.324	0.0419	0.4471	NS						
**General Development**											
ikaros	0.4209	0.095	0.0059	0.7774	NS						
hsp70	0.1472	0.928	0.0268	0.5450	NS						
**Xenobiotic Response**											
cyp1a	**< 0.0001**	**1.045**	**0.3759**	**0.0115**	**0.0009**	**0.1087**	**0.5192**	**0.1415**	**0.4863**	**0.1247**	**0.2976**
cyp1b1	**0.0018**	**1.623**	**0.6166**	**0.0003**	**0.1058**	**9.1303**	**6.8082**	**8.6267**	**6.7251**	**2.9228**	**1.8519**
**References**											
*actc1**	0.2714	-0.038	0.0096	0.7175	NS						
*rps25**	0.0992	-0.140	0.1390	0.1550	NS						
**Morphometrics**											
%Edema	**< 0.0001**	**53.210**	**0.5955**	**0.0005**	**0.0225**	**1.9725**	**1.8185**	**1.8944**	**1.7751**	**0.7028**	**0.6187**
Edema area	**0.0005**	**12864**	**0.6257**	**0.0003**	**0.0532**	**4.6123**	**3.6587**	**4.3773**	**3.6007**	**1.5215**	**1.0735**
SV-YM	0.0011	28.820	0.2278	0.0616	NS						
AVA	**0.0345**	**10.780**	**0.4287**	**0.0059**	**0.1906**	**16.4283**	**11.8957**	**15.491**	**11.772**	**5.1863**	**3.1093**
FS	0.1507	-4.736	0.3069	0.026	ORT						

Source CEWAF

	ANOVA	Regression				[PAH] Extrapolation					
Cardiotoxicity	*p value*	Slope	r2	*p value*	%WAF	∑PAH50	d∑PAH50	∑PAH40	d∑PAH40	∑TAH	d∑TAH
amhc	**< 0.0001**	**-1.763**	**0.5952**	**0.0005**	**0.0217**	**0.6516**	**0.5781**	**0.6299**	**0.5638**	**0.0884**	**0.0517**
cmlc2	**< 0.0001**	**-1.767**	**0.5007**	**0.0022**	**0.0108**	**0.5906**	**0.5253**	**0.5688**	**0.5109**	**0.0765**	**0.0453**
fhl2	**< 0.0001**	**-1.944**	**0.5853**	**0.0006**	**0.0491**	**0.8065**	**0.7120**	**0.7846**	**0.6978**	**0.1187**	**0.0680**
gata4	**< 0.0001**	**-1.022**	**0.6953**	**< 0.0001**	**0.1133**	**1.1693**	**1.0255**	**1.1471**	**1.0116**	**0.1896**	**0.1063**
nkx2.5	0.0002	-0.252	0.2047	0.0785	NS						
nppa	0.0047	-0.604	0.2093	0.0748	NS						
nppb	**0.0007**	**-1.030**	**0.2585**	**0.0443**	**0.0177**	**0.6291**	**0.5586**	**0.6073**	**0.5443**	**0.0840**	**0.0493**
tbx5	**< 0.0001**	**-1.051**	**0.6182**	**0.0003**	**0.0214**	**0.6502**	**0.5768**	**0.6284**	**0.5625**	**0.0881**	**0.0516**
vmhc	**< 0.0001**	**-2.788**	**0.6748**	**< 0.0001**	**0.0310**	**0.7047**	**0.6239**	**0.6829**	**0.6097**	**0.0988**	**0.0573**
**General Development**											
ikaros	0.0852	0.497	0.202	0.0807	NS						
hsp70	0.0446	1.235	0.2462	0.0506	NS						
**Xenobiotic Response**											
cyp1a	0.3407	-0.522	0.04144	0.4495	NS						
cyp1b1	0.0037	0.664	0.2272	0.0619	NS						
**References**											
*actc1**	**< 0.0001**	**-1.237**	**0.8175**	**< 0.0001**	**0.4285**	**2.9502**	**2.5649**	**2.9270**	**2.5523**	**0.5376**	**0.2939**
*rps25**	**< 0.0001**	**-0.660**	**0.7226**	**< 0.0001**	**0.0423**	**0.7684**	**0.6790**	**0.7466**	**0.6648**	**0.1113**	**0.0640**
**Morphometrics**											
%Edema	0.0610	10.720	0.2275	0.0618	NS						
Edema area	0.0955	882.40	0.2098	0.0744	NS						
SV-YM	0.0128	15.720	0.3717	0.0122	ORT						
AVA	0.0197	0.919	0.0173	0.6275	NS						
FS	0.0399	-3.246	0.1836	0.0977	NS						

AW-Source HEWAF

	ANOVA	Regression				[PAH] Extrapolation					
Cardiotoxicity	*p value*	Slope	r2	*p value*	%WAF	∑PAH50	d∑PAH50	∑PAH40	d∑PAH40	∑TAH	d∑TAH
amhc	0.8592	-0.2345	0.0299	0.5222	NS						
cmlc2	0.3135	-0.3750	0.1741	0.1078	NS						
fhl2	0.1570	0.3542	0.1601	0.1246	NS						
gata4	0.0813	0.1632	0.0059	0.7772	NS						
nkx2.5	0.1017	0.4569	0.0360	0.4817	NS						
nppa	**0.0684**	**-0.6205**	**0.3052**	**0.0265**	**0.1353**	**12.6248**	**7.1742**	**11.6529**	**7.1131**	**4.9564**	**1.8779**
nppb	0.0720	-0.6422	0.3369	0.0184	ORT						
tbx5	0.0902	0.1226	0.0034	0.8313	NS						
vmhc	0.1046	0.1390	0.0055	0.7846	NS						
**General Development**											
ikaros	0.7250	-0.1173	0.0227	0.5776	NS						
hsp70	0.1730	1.9190	0.2196	0.0671	NS						
**Xenobiotic Response**											
cyp1a	**< 0.0001**	**1.8500**	**0.7256**	**< 0.0001**	**0.0007**	**0.4314**	**0.2766**	**0.4455**	**0.2690**	**0.2243**	**0.0722**
cyp1b1	**0.0003**	**1.0740**	**0.6379**	**0.0002**	**0.0466**	**4.5834**	**2.6253**	**4.2617**	**2.5995**	**1.8357**	**0.6871**
**References**											
*actc1**	0.0297	-0.1290	0.1026	0.2265	NS						
*rps25**	0.2612	-0.0527	0.0207	0.5949	NS						
**Morphometrics**											
%Edema	**0.0011**	**43.340**	**0.4307**	**0.0058**	**0.0182**	**2.0101**	**1.1696**	**1.8965**	**1.1551**	**0.8370**	**0.3060**
Edema area	**< 0.0001**	**9426.0**	**0.6815**	**< 0.0001**	**0.0291**	**3.0031**	**1.7313**	**2.8092**	**1.7125**	**1.2224**	**0.4531**
SV-YM	**< 0.0001**	**40.000**	**0.538**	**0.0012**	**0.0175**	**1.9475**	**1.1342**	**1.8390**	**1.1200**	**0.8127**	**0.2967**
AVA	0.0566	16.590	0.6394	0.0002	ORT						
FS	**0.0229**	**-7.2610**	**0.3365**	**0.0185**	**0.1160**	**10.8759**	**6.1849**	**10.045**	**6.1315**	**4.2777**	**1.6189**

AW-Source CEWAF

	ANOVA	Regression				[PAH] Extrapolation					
Cardiotoxicity	*p value*	Slope	r2	*p value*	%WAF	∑PAH50	d∑PAH50	∑PAH40	d∑PAH40	∑TAH	d∑TAH
amhc	**< 0.0001**	**-2.096**	**0.5440**	**0.0011**	**0.0099**	**0.5491**	**0.2437**	**0.5060**	**0.2383**	**0.2438**	**0.0873**
cmlc2	**< 0.0001**	**-1.997**	**0.5933**	**0.0005**	**0.0034**	**0.2606**	**0.1104**	**0.2306**	**0.1057**	**0.1282**	**0.0504**
fhl2	**< 0.0001**	**-2.986**	**0.6856**	**< 0.0001**	**0.0260**	**1.2580**	**0.5715**	**1.1826**	**0.5642**	**0.5280**	**0.1779**
gata4	**< 0.0001**	**-1.382**	**0.5615**	**0.0008**	**0.0053**	**0.3434**	**0.1486**	**0.3096**	**0.1438**	**0.1614**	**0.0610**
nkx2.5	< 0.0001	-0.254	0.1332	0.1645	NS						
nppa	**< 0.0001**	**-1.688**	**0.5755**	**0.0007**	**0.0148**	**0.7637**	**0.3429**	**0.7108**	**0.3369**	**0.3298**	**0.1147**
nppb	**< 0.0001**	**-2.569**	**0.6732**	**< 0.0001**	**0.0132**	**0.6930**	**0.3102**	**0.6433**	**0.3044**	**0.3015**	**0.1057**
tbx5	**< 0.0001**	**-1.076**	**0.5321**	**0.0013**	**0.0128**	**0.6747**	**0.3018**	**0.6258**	**0.2960**	**0.2942**	**0.1033**
vmhc	**< 0.0001**	**-3.501**	**0.6609**	**0.0001**	**0.0093**	**0.5217**	**0.2311**	**0.4799**	**0.2257**	**0.2329**	**0.0838**
**General Development**											
ikaros	0.2917	0.335	0.0805	0.2869	NS						
hsp70	0.0252	2.170	0.4371	0.0053	ORT						
**Xenobiotic Response**											
cyp1a	0.0107	-1.080	0.1667	0.1164	NS						
cyp1b1	0.0034	0.931	0.2423	0.0528	NS						
**References**											
*actc1**	**0.0635**	**-1.486**	**0.4198**	**0.0066**	**0.1283**	**5.7613**	**2.6536**	**5.4807**	**2.6341**	**2.3327**	**0.7536**
*rps25**	**0.1139**	**-0.635**	**0.2579**	**0.0446**	**0.0685**	**3.1280**	**1.4361**	**2.9674**	**1.4237**	**1.2774**	**0.4170**
**Morphometrics**											
%Edema	**0.0170**	**43.260**	**0.5266**	**0.0015**	**0.1523**	**6.8180**	**3.1422**	**6.4893**	**3.1199**	**2.7562**	**0.8887**
Edema area	0.1518	4730.0	0.3097	0.0252	ORT						
SV-YM	**< 0.0001**	**39.760**	**0.7352**	**< 0.0001**	**0.0572**	**2.6310**	**1.2063**	**2.4930**	**1.1953**	**1.0782**	**0.3534**
AVA	0.1703	6.337	0.3039	0.0269	ORT						
FS	0.2404	-1.465	0.01943	0.6067	NS						

Slick A HEWAF

	ANOVA	Regression				[PAH] Extrapolation					
Cardiotoxicity	p value	Slope	r2	p value	%WAF	∑PAH50	d∑PAH50	∑PAH40	d∑PAH40	∑TAH	d∑TAH
amhc	0.8029	-0.171	0.0791	0.3300	NS						
cmlc2	0.5163	-0.178	0.1294	0.2065	NS						
fhl2	0.3623	0.047	0.0144	0.6701	NS						
gata4	0.8640	0.041	0.0101	0.7329	NS						
nkx2.5	0.2260	-0.233	0.1381	0.1907	NS						
nppa	0.5793	0.087	0.0239	0.5974	NS						
nppb	0.8526	-0.038	0.0021	0.8771	NS						
tbx5	0.3897	0.187	0.0813	0.3232	NS						
vmhc	0.3445	0.036	0.0017	0.8890	NS						
**General Development**											
ikaros	0.4900	-0.202	0.0336	0.5129	NS						
hsp70	**0.0048**	**1.698**	**0.6901**	**0.0001**	**0.112696**	**4.5919**	**2.3584**	**3.8259**	**1.9616**	**2.0635**	**1.0858**
**Xenobiotic Response**											
cyp1a	**< 0.0001**	**1.951**	**0.8932**	**< 0.0001**	**0.003632**	**0.6656**	**0.4125**	**0.2912**	**0.2230**	**0.0793**	**0.0597**
cyp1b1	0.8660	-0.131	0.0193	0.6216	NS						
**References**											
*actc1**	0.7168	0.094	0.0762	0.3193	NS						
*rps25**	0.6742	-0.046	0.0383	0.4844	NS						
**Morphometrics**											
%Edema	**0.0025**	**19.110**	**0.5955**	**0.0012**	**0.026565**	**1.4911**	**0.8216**	**1.0345**	**0.5886**	**0.4965**	**0.2754**
Edema area	0.0321	329.70	0.06539	0.3391	NS						
SV-YM	0.011	1.890	0.01502	0.6511	NS						
AVA	0.0385	-7.219	0.1477	0.1416	NS						
FS	0.0567	1.834	0.1528	0.1344	NS						

Slick A CEWAF

	ANOVA	Regression				[PAH] Extrapolation					
Cardiotoxicity	p value	Slope	r2	p value	%WAF	∑PAH50	d∑PAH50	∑PAH40	d∑PAH40	∑TAH	d∑TAH
amhc	0.0148	0.6264	0.06897	0.3443	NS						
cmlc2	0.3188	0.193	0.04708	0.4196	NS						
fhl2	0.4330	-0.2955	0.09916	0.253	NS						
gata4	**0.0281**	**0.9658**	**0.5651**	**0.0012**	**6.2675**	-	-	-	-	-	-
nkx2.5	0.1662	0.1219	0.06346	0.3466	NS						
nppa	0.4499	-0.1792	0.08294	0.2794	NS						
nppb	0.0858	-0.1692	0.05466	0.3835	NS						
tbx5	0.0672	0.468	0.2231	0.0754	NS						
vmhc	0.4882	0.1907	0.03259	0.5034	NS						
**General Development**											
ikaros	0.0787	0.1002	0.04697	0.4378	NS						
hsp70	0.1368	0.8684	0.1085	0.2305	NS						
**Xenobiotic Response**											
cyp1a	**< 0.0001**	**2.147**	**0.8864**	**< 0.0001**	**0.0465**	-	-	-	-	-	-
cyp1b1	**< 0.0001**	**1.649**	**0.8821**	**< 0.0001**	**0.8562**	-	-	-	-	-	-
**References**											
*actc1**	0.0314	-0.6734	0.5146	0.0018	ORT						
*rps25**	0.0836	-0.3638	0.2804	0.0349	ORT						
**Morphometrics**											
%Edema	**0.0001**	**26.69**	**0.5254**	**0.0015**	**1.3843**	-	-	-	-	-	-
Edema area	**0.0029**	**3724**	**0.3889**	**0.0098**	**0.7346**	-	-	-	-	-	-
SV-YM	**0.0082**	**19.02**	**0.3931**	**0.0093**	**0.9119**	-	-	-	-	-	-
AVA	**0.0024**	**5.762**	**0.6464**	**0.0002**	**1.7016**	-	-	-	-	-	-
FS	**0.0127**	**-2.918**	**0.4878**	**0.0026**	**0.5194**	-	-	-	-	-	-

Indicators with significant log- linear regression ($p < 0.05$) were extrapolated from threshold %WAF dilution to each of four constituent PAH measurements (ΣPAH40, sum of measured 40 polycyclic aromatic hydrocarbons, PAHs; ΣTAH, sum of measured tricyclic aromatic hydrocarbons TAHs; dΣPAH, sum of modeled dissolved total PAHs; dΣTAH, sum of modeled dissolved TAHs: see Methods; Supplemental Materials: Figure S6). One-way ANOVA and log-linear regression significance determined using JMP (Version 10) and PRISM (Version 5) software, respectively, using α = 0.05. *NS*, Non-significant log-linear regression ($p > 0.05$); *ORT*, significant log-linear regression ($p < 0.05$) but effective %WAF above range tested.

Table S2. Summary of time-course two-way ANOVAs.

				Treatment Effects		
**Gene**	**F Ratio**	***p* Value**	**DF**	**Time**	**Treatment**	**Age*Treatment**
amhc	5.304	0.0041*	5,17	0.0007*	0.5820	0.0931
cmlc2	8.777	0.0003*	5,17	0.1925	0.0011*	0.0002*
fhl2	15.990	<.0001*	5,17	<.0001*	<.0001*	0.0004*
gata4	4.401	0.0094*	5,17	0.0012*	0.4960	0.3598
nkx2.5	9.676	0.0002*	5,17	0.9779	0.0044*	<.0001*
nppa	8.010	0.0005*	5,17	0.172	0.0015*	0.0011*
nppb	1.929	0.1421	5,17	0.2628	0.0331*	0.5794
tbx5	11.648	<.0001*	5,17	<.0001*	0.4472	0.0046*
vmhc	16.924	<.0001*	5,17	<.0001*	0.0008*	0.0013*
hsp70	0.898	0.5047	5,17	0.725	0.1774	0.3694

Summary of two-way ANOVAs run on molecular indicator expression data (log_2-transformed fold-change) collected for pools of larval mahi mahi sampled at three discrete exposure durations (48, 53.5 and 58 hours post-exposure; see Methods; Figure 4). Treatment effects considered by two-way ANOVA were Age, Treatment and Age*Treatment interaction. Significance of two-way ANOVA and Tukey-Kramer post-hoc analyses (Figure 7) were determined using JMP (Version 10) with $\alpha = 0.05$. Asterisks denote significant two-way ANOVA ($p < 0.05$).

Table S3. Quantitative PCR (qPCR) primer sequences and amplicon identity verification.

Gene	Primers (5' - 3')	GenBank	Species	E Value
amhc	F - TCACCAACAACCCGTATGACT<br>R - ACGCCCATCTTCTCCTCTG	JX190488	*Coryphaena hippurus*	1.00E-42
cmlc2	F - GACCAAGACAGAGACGGTGTTA<br>R - GCATCTCATCCAACTCCTCGT	NM_001145995	*Oryzias latipes*	1.00E-21
fhl2	F - GCTTCAAGTGCTTCCAGTGC<br>R - GCAGGTGAAGCAGGTCTCAT	DQ225183	*Sparus aurata*	3.00E-80
gata4	F - AGAGTGTGTGAACTGCGGG<br>R - TGACAGTTGGTGCAGGACAG	NM_001279627	*Oreochromis niloticus*	3.00E-61
nkx2.5	F - CAAGCAGCAGAGGTACCTGT<br>R - CAGATCTTCACCTGGGTCGG	NM_001104912	*Oryzias latipes*	4.00E-15
nppa	F - CAGCAGGAGGACTTTGAAGA<br>R - ATCAGGGGTTCTTGATTTCC	AB162777	*Cottus kazika*	6.00E-37
nppb	F - AAAGAGTCCCTCTGGAGCAG<br>R - CAGGTTCTTGGCTGAGAGAA	AB162778	*Cottus kazika*	3.00E-14
tbx5	F - GGTCACAGGCCTCAATCCAA<br>R - AGAGTCCGGGTGAACGTAGA	NM_001201500	*Oryzias latipes*	8.00E-42
vmhc	F - CCAAAACCATCAGGAATGAC<br>R - TCAGCTCTTCAGCATCATTG	NM_001112733	*Danio rerio*	7.00E-78
cyp1a	F - TGTCTTCATCAATCAGTGGCAG<br>R - CGTTTGTGCTTCATTGTGAGAC	EU163982	*Pagrus major*	8.00E-28
cyp1b1	F - CTCGTGGTGTCCACTGCCA<br>R - CCAGGGCATAACGTCCAC	AJ249074	*Pleuronectes platessa*	2.00E-54
hsp70	F - AGATCGAGAGGATGGTGCA<br>R - CCAGCTGGTTGTTCTCCAG	AB436470	*Seriola quinqueradiata*	6.00E-77
ikaros	F - CAACCAGAATGAGCAGAGGG<br>R - CTTCTGTGGCATAGTGCTCT	GQ466344	*Cynoglossus semilaevis*	2.00E-40
actc1	F - TATGCCAACAACGTGCTCTC<br>R - CACCGATCCAGACGGAGTAT	NM_131591	*Danio rerio*	6.00E-56
rps25	F - GACAAGGCGACCTACGACAA<br>R - CCACGGATCTTCAGCCTCTC	AB291578	*Solea senegalensis*	9.00E-24

Quantitative PCR (qPCR) primer oligonucleotides and nucleotide BLAST results for sequenced qPCR amplicons of candidate molecular indicators. E value provides confidence in molecular indicator annotations. GenBank accession number and corresponding species are provided.
